# The mechanism of sirtuin 2–mediated exacerbation of alpha-synuclein toxicity in models of Parkinson disease

**DOI:** 10.1371/journal.pbio.2000374

**Published:** 2017-03-03

**Authors:** Rita Machado de Oliveira, Hugo Vicente Miranda, Laetitia Francelle, Raquel Pinho, Éva M. Szegö, Renato Martinho, Francesca Munari, Diana F. Lázaro, Sébastien Moniot, Patrícia Guerreiro, Luis Fonseca, Zrinka Marijanovic, Pedro Antas, Ellen Gerhardt, Francisco Javier Enguita, Bruno Fauvet, Deborah Penque, Teresa Faria Pais, Qiang Tong, Stefan Becker, Sebastian Kügler, Hilal Ahmed Lashuel, Clemens Steegborn, Markus Zweckstetter, Tiago Fleming Outeiro

**Affiliations:** 1 CEDOC, NOVA Medical School, Faculdade de Ciências Médicas, Universidade NOVA de Lisboa, Lisboa, Portugal; 2 Department of Neurodegeneration and Restorative Research, University Medical Center Göttingen, Göttingen, Germany; 3 Faculty of Medicine, University of Porto, Porto, Portugal; 4 Instituto de Medicina Molecular, Faculdade de Medicina, Universidade de Lisboa, Lisboa, Portugal; 5 Department for NMR-based Structural Biology, Max Planck Institute for Biophysical Chemistry, Göttingen, Germany; 6 German Center for Neurodegenerative Diseases (DZNE), Göttingen, Germany; 7 Department of Biochemistry, University of Bayreuth, Bayreuth, Germany; 8 Laboratory of Molecular and Chemical Biology of Neurodegeneration, Swiss Federal Institute of Technology Lausanne (EPFL), Lausanne, Switzerland; 9 Laboratório de Proteómica, Departamento de Genética Humana, Instituto Nacional de Saúde Dr. Ricardo Jorge, Lisboa, Portugal; 10 USDA/ARS Children's Nutrition Research Center, Department of Pediatrics, Baylor College of Medicine, Houston, Texas, United States of America; 11 Department of Neurology, University Medical Center Göttingen, University of Göttingen, Göttingen, Germany; 12 Max Planck Institute for Experimental Medicine, Göttingen, Germany; 13 Center for Biostructural Imaging of Neurodegeneration, University Medical Center Göttingen, University of Göttingen, Göttingen, Germany; University College London, UNITED KINGDOM

## Abstract

Sirtuin genes have been associated with aging and are known to affect multiple cellular pathways. Sirtuin 2 was previously shown to modulate proteotoxicity associated with age-associated neurodegenerative disorders such as Alzheimer and Parkinson disease (PD). However, the precise molecular mechanisms involved remain unclear. Here, we provide mechanistic insight into the interplay between sirtuin 2 and α-synuclein, the major component of the pathognomonic protein inclusions in PD and other synucleinopathies. We found that α-synuclein is acetylated on lysines 6 and 10 and that these residues are deacetylated by sirtuin 2. Genetic manipulation of sirtuin 2 levels in vitro and in vivo modulates the levels of α-synuclein acetylation, its aggregation, and autophagy. Strikingly, mutants blocking acetylation exacerbate α-synuclein toxicity in vivo, in the substantia nigra of rats. Our study identifies α-synuclein acetylation as a key regulatory mechanism governing α-synuclein aggregation and toxicity, demonstrating the potential therapeutic value of sirtuin 2 inhibition in synucleinopathies.

## Author summary

Parkinson disease is an age-associated neurodegenerative disorder characterized by the loss of dopamine-producing neurons from a region in the brain known as the substantia nigra and by the accumulation of the protein alpha-synuclein in intracellular clumps called inclusions. Whether these inclusions are the cause or a consequence of the pathological processes is still unclear. Sirtuin proteins are considered master regulators of the ageing process and have previously been associated with neurodegeneration. In this study, we investigated the interplay between sirtuin 2 and alpha-synuclein in order to dissect the molecular mechanisms associated with protection against alpha-synuclein toxicity. We found that sirtuin 2 interacted with and removed acetyl groups from alpha-synuclein. By decreasing the levels of sirtuin 2, or by expressing mutant versions of alpha-synuclein that modulate its acetylation status, we found that acetylation reduces the aggregation of alpha-synuclein and its cytotoxicity in vitro. Next, we evaluated whether genetic inhibition of sirtuin 2 could prevent the deleterious effects of alpha-synuclein in vivo and found that, in two different models of Parkinson disease, deletion of sirtuin 2 was neuroprotective. Our data therefore suggest that strategies aimed at decreasing sirtuin 2 activity might prove valuable therapeutic avenues for intervention in Parkinson disease and other synucleinopathies.

## Introduction

Sirtuins are NAD^+^-dependent deacylases and lifespan determinants in several model organisms. Sirtuin proteins have been implicated in neurodegenerative disorders, conditions that are strongly associated with aging [1,2]. In mammals, there are seven members of the sirtuin (SIRT) family: SIRT1–SIRT7. SIRT2 is the most abundant sirtuin in the brain and its levels increase with aging [3]. Interestingly, SIRT2 emerged as a potential culprit in Parkinson disease (PD) pathology, as we showed that SIRT2 modulates α-synuclein (aSyn) aggregation and toxicity [4]. Pharmacological inhibition of SIRT2 ameliorates aSyn-mediated toxicity in cell models and in vivo (in a *Drosophila* model of PD), but the molecular mechanisms underlying this effect remain unclear [4]. aSyn is the main constituent of Lewy bodies, protein inclusions typically found in the brains of PD patients [5], and is therefore a central protein in PD. Interestingly, the balance between acetylation and deacetylation is altered in both aging and neurodegeneration, and a link between acetylation of nonhistone proteins and neuroprotection has recently emerged [6]. Given that SIRT2 is a deacetylase and that modulating its activity affects aSyn aggregation and toxicity, we hypothesized that SIRT2 may induce neurodegeneration by modulating aSyn acetylation, rendering it more prone to aggregate and to be cytotoxic. Here, we provide detailed insight into the mechanism through which SIRT2 modulates aSyn toxicity and demonstrate that acetylation on lysine (K)6 and K10 might be used as targets for therapeutic intervention in PD and in other synucleinopathies.

## Results

### SIRT2 interacts with and deacetylates aSyn

**Fig 1:**
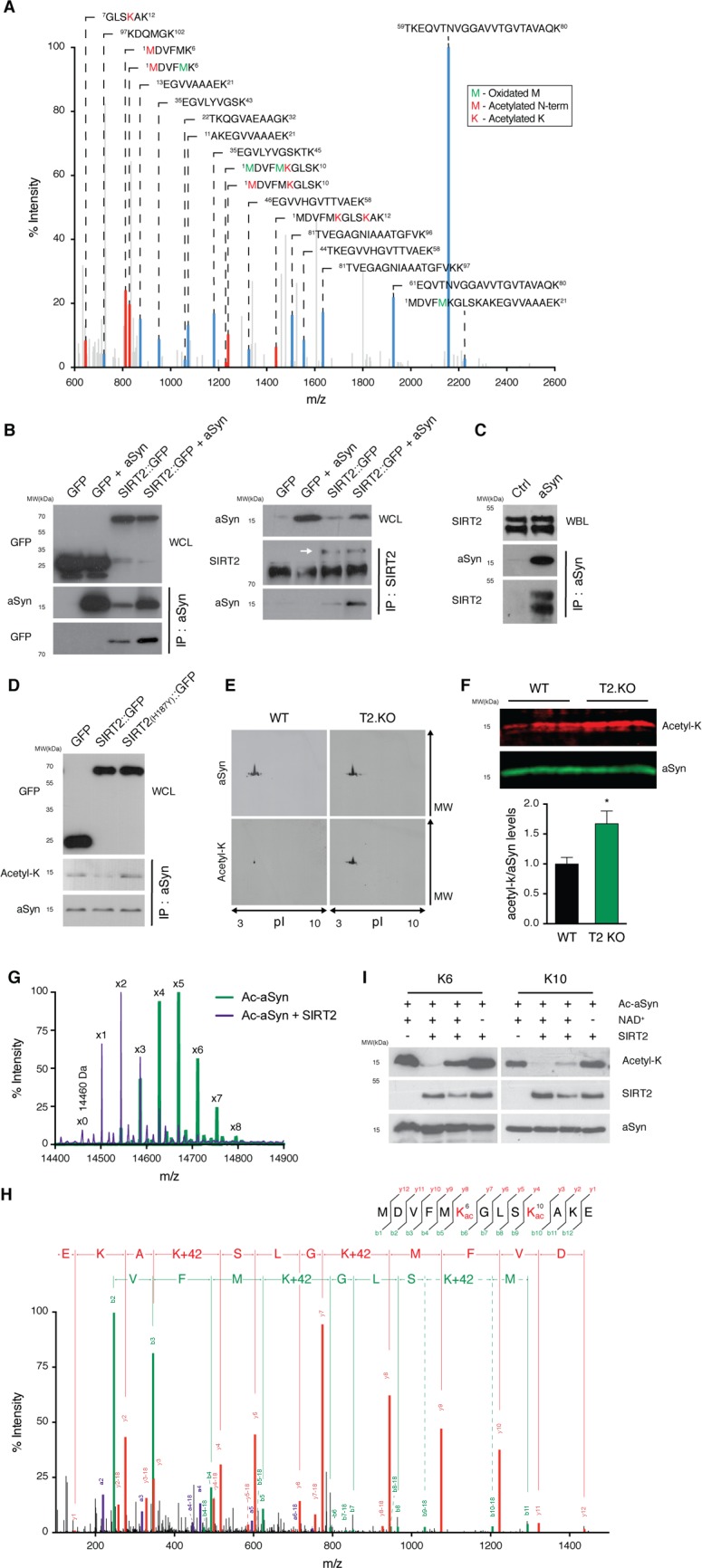
SIRT2 interacts with aSyn and deacetylates lysine 6 and 10. **(A)** Detection of aSyn acetylation on lysine 6 (K6) and lysine 10 (K10) by peptide mass fingerprinting analysis in total mouse brain lysates. Spectrum shows acetylated peptides in red and aSyn peptides in blue. The corresponding peptide sequences are shown (red residues are acetylated and green residues are oxidated). **(B)** Human HEK293T cells expressing the indicated proteins were lysed and immunoprecipitated (IP) with anti-aSyn (left panels) or anti-SIRT2 polyclonal antibodies (right panel). The whole-cell lysates (WCL) and immunoprecipitation samples were probed for GFP (left panels) or aSyn (right panels). **(C)** Mouse whole-brain lysates (WBL) were lysed and IP with anti-aSyn polyclonal antibody. The IP sample was probed with anti-SIRT2 and anti-aSyn. Rabbit IgG was used as a negative control for the IP sample. **(D)** Immunoprecipitations were probed with an anti–acetyl-lysine antibody in cells expressing either SIRT2 or the SIRT2-H187Y inactive mutant. **(E)** Immunoblot of thermoenriched aSyn from mouse-brain lysate probed for acetyl-lysine and aSyn. **(F)** Brain protein extracts from WT and SIRT2 knockout (T2.KO) mice were separated by SDS-PAGE and immunoblotted with antibodies against acetyl-lysine and aSyn (*n* = 3). The ratio of acetyl-lysine to aSyn is presented. *p < 0.05, unpaired *t* test with equal standard deviation (SD). **(G)** Overlay of the deconvoluted intact protein mass spectra obtained from chemically acetylated aSyn (theoretical mass 14,460 Da) in buffer (green) and treated with SIRT2 (purple). The observed masses of the different species correlate with the presence of multiple acetyl modifications (+42 Da), ranging from 2 to 8 before treatment with SIRT2 and from 0 to 6 after deacetylation with SIRT2. **(H)** Mass spectrometry fragmentation analysis of a peptide from aSyn carrying acetylations at K6 and K10. Red peaks correspond to y-ion series, green peaks to b-ion series, and purple peaks to a-ion series. Corresponding amino acids to mass intervals of y-ion and b-ion series are represented (red and green, respectively). **(I)** Semisynthetic aSyn acetylated at K6 and K10 were incubated with increasing amounts of recombinant SIRT2 in the presence or absence of NAD at 37°C for 3 h. Proteins were probed for acetyl-lysine residues, aSyn, and SIRT2. All images are representative out of three independent experiments. Data in S1 Data.

To determine the mechanism of SIRT2-mediated protection against aSyn toxicity and aggregation, we hypothesized that aSyn might be acetylated and that this could be a substrate for SIRT2. Thus, we first evaluated if aSyn was acetylated in mouse brain. To this purpose, endogenous aSyn was thermoenriched from the brain of wild-type (WT) mice and evaluated by mass spectrometry (MS) as we previously described [7]. Notably, we confirmed that aSyn is ubiquitously acetylated at the N-terminus (Fig 1A and S1 Table) [8]. In addition, we identified K6 and K10, in the conserved N-terminal region, as aSyn acetylation sites (Fig 1A and S1 Table). Next, we investigated whether SIRT2 and aSyn interact, as this would be an indication that perhaps aSyn is a substrate for SIRT2. We coexpressed SIRT2 (tagged with green fluorescent protein [GFP]) with aSyn in human embryonic kidney (HEK) 293T cells and immunoprecipitated aSyn or SIRT2. Using immunoblot analyses, we confirmed the coimmunoprecipitation of aSyn with SIRT2, demonstrating that both proteins interacted (Fig 1B). To confirm the interaction in a physiologically relevant context (i.e., without overexpressing the proteins), we immunoprecipitated aSyn from mouse brain extracts and, in agreement with the results obtained in transfected HEK cells, we found that SIRT2 coimmunoprecipitated with aSyn (Fig 1C).

Next, we investigated whether acetylated aSyn is a substrate of SIRT2 deacetylase activity. We transiently expressed aSyn together with SIRT2 or with the enzymatically inactive SIRT2-H187Y mutant in HEK cells and measured the levels of aSyn acetylation after immunoprecipitation and immunoblotting with an anti-acetyl–lysine antibody. We found that SIRT2, but not the inactive SIRT2-H187Y mutant, reduced the levels of acetylated aSyn (Fig 1D). We then assessed the levels of aSyn acetylation in the brains of SIRT2 knockout (T2.KO) mice. Consistently, we found that deletion of SIRT2 leads to aSyn hyperacetylation (Fig 1E and 1F). We also observed an age-associated reduction in the levels of aSyn acetylation in T2.KO mice but not in WT animals (S1 Fig).

To further investigate the ability of SIRT2 to deacetylate aSyn, we performed in vitro deacetylation assays. Chemical acetylation of aSyn yielded two to eight acetylations per protein molecule, with a distribution maximum of five acetyl modifications (Fig 1G). Peptide MS revealed that three of the five KxK motifs in aSyn, namely K21/23, K32/34, and K58/60, showed little or no acetylation, while all other lysines—including K6 and K10—were acetylated efficiently (Fig 1H, S2 Fig). In vitro treatment of acetylated aSyn with SIRT2 and NAD^+^, its cofactor, caused efficient deacetylation, as demonstrated by the shift of the modification peak maximum from five to two acetyl groups (Fig 1G). We also investigated the specific SIRT2-dependent deacetylation of aSyn on lysines K6 and K10. For this, we generated acetylated forms of aSyn specifically on residues K6 or K10 (acK6 and acK10) using our previously established semisynthetic approach [9,10]. We found that SIRT2 indeed deacetylates both acK6 and acK10 in a dose-dependent and in an NAD^+^-dependent manner, with similar efficiency (Fig 1I). In conclusion, we identified K6 and K10 as aSyn acetylation sites that are targeted by SIRT2.

### SIRT2 KD suppresses aSyn aggregation and toxicity

**Fig 2:**
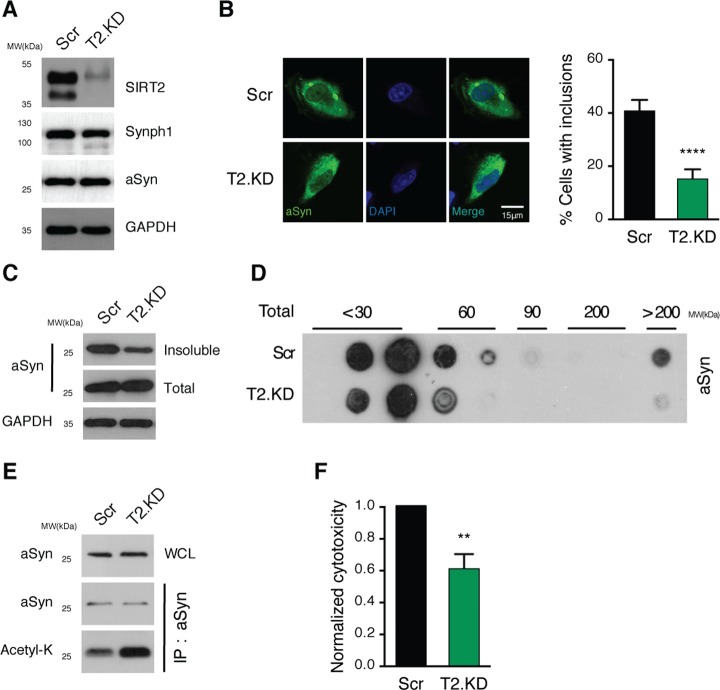
SIRT2 regulates aSyn aggregation and toxicity. **(A)** H4 cells were infected with lentiviruses encoding shRNAs against SIRT2 (T2.KD) or scramble shRNA (Ctrl) and selected with puromycin. Cells were then cotransfected with SynT and synphilin-1 (Synph1). SIRT2, synphilin-1, aSyn, and GAPDH levels were assessed by immunoblot analyses. **(B)** Ctrl and T2.KD cells transiently expressing SynT and synphilin-1 for 48 h were processed for immunocytochemistry (ICC) (aSyn, green). Data show percentage of cells with aSyn inclusions (*n* = 3). Scale bar 15 μm. **(C)** Triton X-100 insoluble and total fractions of cells as in (B) probed for aSyn and GAPDH. **(D)** Native protein extracts from H4 cells as in (B) were separated on a sucrose gradient. Fractions were immunoblotted and probed for aSyn. **(E)** Anti-aSyn IP from cells as in (B). Fractions were immunoblotted and probed for acetyl-lysine and aSyn. **(F)** Toxicity of Ctrl and T2.KD measured by lactate dehydrogenase (LDH) release assay (*n* = 3). Data in all panels are average ± SD, ** *p* < 0.01, **** *p* < 0.0001. For (B) and (F), unpaired, two-tailed *t* test with equal SD. Data in S1 Data.

Given that we demonstrated that aSyn acetylation can be modulated by SIRT2, we investigated whether SIRT2 modulates aSyn aggregation. To that purpose, we decreased SIRT2 levels using short hairpin RNA (shRNA)-mediated knockdown in an established cellular model of PD in which aSyn aggregation is recapitulated. This model consists of the coexpression of a C-terminally modified form of aSyn (SynT) that increases its aggregation propensity, and synphilin-1 (an aSyn interactor that potentiates its aggregation) in H4 cells [11,12]. Upon SIRT2 knockdown (T2.KD), we detected comparable levels of SynT and synphilin-1 as in cells transduced with scramble shRNA (Scr) (Fig 2A). To quantify the effect on aSyn aggregation, T2.KD and Scr cells were analyzed by immunocytochemistry (ICC) 48 h posttransfection. Remarkably, T2.KD decreased the percentage of cells displaying SynT-positive inclusions to half of that in control cells (Fig 2B). Biochemically, we confirmed that T2.KD increased SynT triton X-100 solubility (Fig 2C) and decreased the levels of higher-molecular-weight (MW) oligomeric species, as assessed by sucrose gradient separation (Fig 2D). Next, we evaluated the levels of acetylated aSyn in the same cell-based aggregation model. By immunoprecipitating SynT, we observed that T2.KD increased the levels of acetylated aSyn (Fig 2E). Importantly, concomitantly with the decrease in SynT aggregation and hyperacetylation, T2.KD also decreased the cytotoxicity of SynT, as measured by the levels of lactate dehydrogenase (LDH) release from cells (Fig 2F).

### K6 and K10 acetylation modulate aSyn aggregation and membrane-binding ability

**Fig 3:**
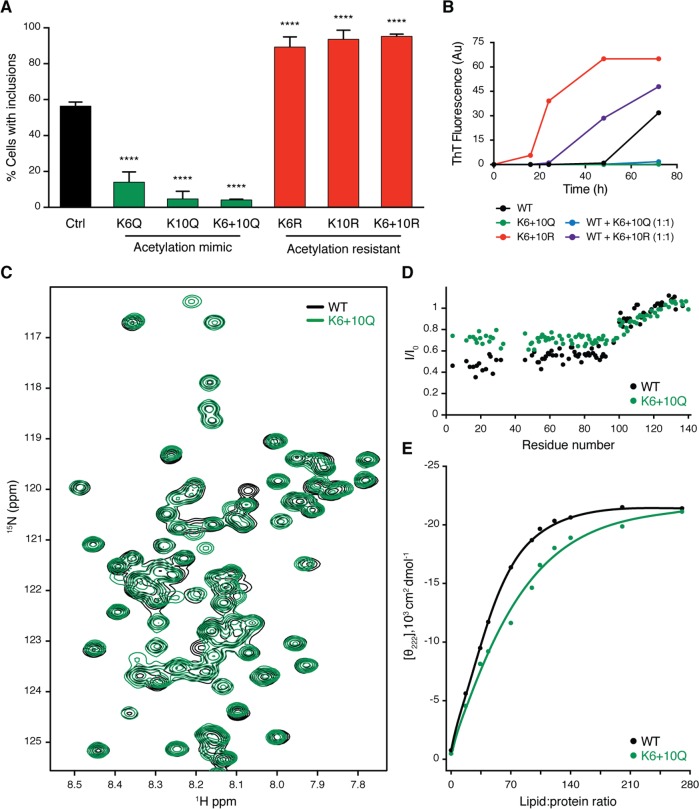
aSyn aggregation is modulated by acetylation. **(A)** H4 cells were cotransfected with WT, acetylation-resistant mutants (K6R, K10R, K6+10R), or acetylation-mimicking mutants (K6Q, K10Q, K6+10Q) of SynT together with synphilin-1. 48 h after transfection, cells were processed for ICC and the percentage of cells with inclusions was determined (*n* = 3). Data in panels are average ± SD, **** *p* < 0.0001, ordinary one-way ANOVA with Tukey’s multiple comparisons test. **(B)** Oligomerization kinetics of recombinant aSyn assessed by Thioflavin-T reaction. WT, K6+10Q, K6+10R, and a mixture of 1:1 of WT with K6+10Q or K6+10R were evaluated. **(C)** Superposition of 2D ^1^H-^15^N HSQC nuclear magnetic resonance (NMR) spectra of recombinant ^15^N-labelled aSyn WT (black), K6+10Q (green). **(D)** Residue-specific changes in ^1^H-^15^N HSQC signal intensities of aSyn WT (black) and aSyn K6+10Q (green) upon addition of small unilamellar vesicles (SUVs) formed by POPC:POPA (1:1 molar ratio). The aSyn-to-lipid molar ratio was 1:100. **(E)** Estimation of the binding affinity of aSyn to POPC:POPA SUVs from circular dichroism. Variations in absorption at 222 nm are plotted as a function of lipid:protein molar ratio. Calculated affinities are 57 ± 13 and 71 ± 16 μM for aSyn WT (black) and aSyn K6+10Q (green), respectively. Data in S1 Data.

To determine whether K6 and K10 acetylation modulated aSyn aggregation, we generated aSyn mutants in which both lysine residues were replaced by either arginine residues (KR) to mimic acetylation-resistant aSyn or by glutamine residues (KQ) to mimic constitutively acetylated aSyn [13]. Using the cell model of aSyn aggregation described above, we observed that while the acetylation-resistant mutant KR promoted SynT aggregation, the acetylation mimic mutant KQ prevented it (Fig 3A).

To assess if the observed phenotype was due to an intrinsic modulation of aSyn aggregation, we investigated the oligomerization kinetics of recombinant mutant aSyn, as previously described [14]. The kinetics of aSyn fibril formation was monitored using thioflavin T (ThT). For the WT aSyn, we observed the expected increase in ThT fluorescence signal, confirming the formation of amyloid-like fibrils (Fig 3B). Interestingly, we observed that while acetylation-resistant mutants increased, acetylation-mimic mutants prevented aSyn oligomerization (Fig 3B). Moreover, when coincubated with WT aSyn, acetylation-resistant mutants also increased, while acetylation-mimic mutants prevented WT aSyn oligomerization (Fig 3B).

We then investigated the effects of aSyn acetylation on its structure, using nuclear magnetic resonance (NMR) spectroscopy, using again recombinant KR and KQ aSyn mutants produced in *Escherichia coli*, as above. Two-dimensional ^1^H-^15^N correlation spectra of soluble aSyn and its mutants showed a narrow spread of NMR resonances, demonstrating that both WT and acetylation-resistant and/or acetylation-mimic mutants are intrinsically disordered (Fig 3C and S3A Fig). In addition, a high degree of NMR signal overlap between the WT and mutant proteins suggested that the mutations do not strongly perturb the conformational ensemble of aSyn in solution (Fig 3C and S3A Fig).

Next, we probed the impact of acetylation-resistant and acetylation-mimic mutants on the ability of aSyn to bind to lipid membranes, a property thought to be associated with the physiological function of the protein. In the presence of small unilamellar vesicles (SUVs), aSyn constantly exchanges between the soluble disordered state and the helical, membrane-bound structure [15]. Because of this exchange process, aSyn residues involved in membrane-binding experience NMR line broadening [15], in which residues with stronger binding generally experience more broadening. We detected the residue-specific NMR signal attenuation, which is typically observed for aSyn in the presence of SUVs: the N-terminal 100 residues are strongly attenuated—in particular the N-terminal 20–30 residues, which anchor aSyn to the membrane surface [16]—while the C-terminal domain of aSyn binds only very weakly to POPC:POPA membranes (Fig 3D and S3B Fig). In the case of the acetylation-resistant mutant (aSyn KR), a very similar profile was observed, demonstrating that replacement of lysine residues at positions 6 and 10 with arginine does not perturb the exchange of aSyn between the solution and membrane-bound state (S3B Fig). In contrast, addition of POPC:POPA SUVs to the acetylation-mimic mutant (aSyn KQ) resulted in less signal attenuation, and thus higher NMR signal intensities, in the N-terminal 100 residues when compared to the WT protein (Fig 3D). The strongest difference was observed for the N-terminal 20–30 residues. Because of the dynamic nature of the aSyn–membrane interaction, the overall affinity of aSyn for POPC:POPA membranes was only slightly attenuated, as estimated by circular dichroism (Fig 3E).

### aSyn acetylation modulates autophagy

**Fig 4:**
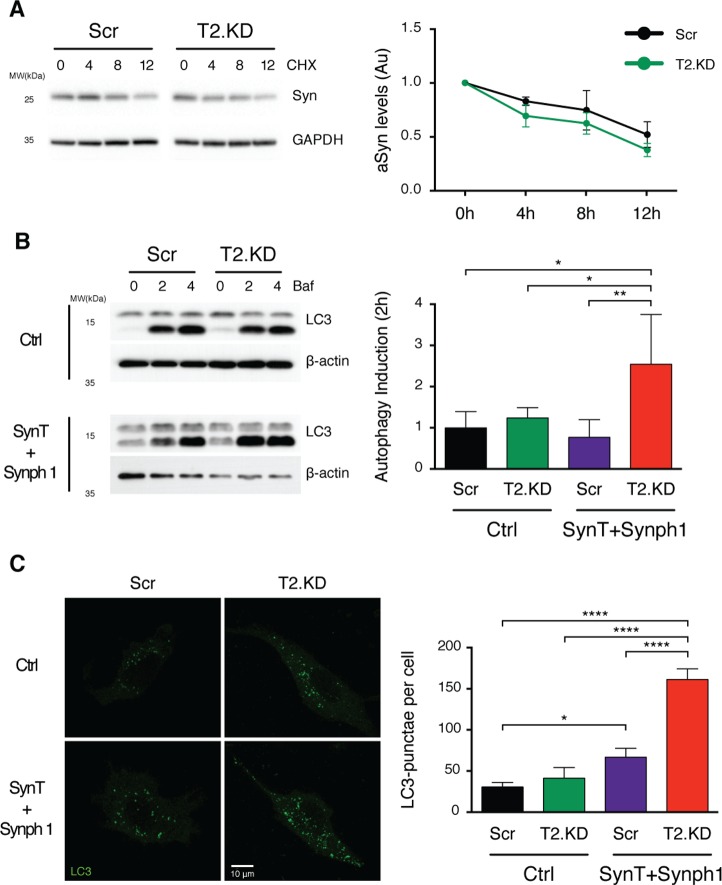
Acetylation of aSyn enhances the clearance of aSyn inclusions by autophagy. **(A)** H4 cells (control and T2.KD) transiently expressing SynT and synphilin-1 were treated with cycloheximide (CHX) for 4, 8, and 12 h. Protein extracts were then separated on SDS-PAGE and probed for aSyn and GAPDH for normalization (at least *n* = 3). **(B)** Cells as in (A) were treated with bafilomycin A1 (BafA) for 2 and 4 h. Protein extracts were probed for LC3 and β-actin. LC3-II levels were normalized to β-actin, and the difference between BafA treatment for 2 h and vehicle (0 h) treatment was calculated (at least *n* = 3). **(C)** Cells as in (A) were processed for ICC (LC3, green). Number of LC3 puncta per cell were counted (*n* = 3). Data in all panels are average ± SD, * *p* < 0.05, ** *p* < 0.01. For (B) and (C), ordinary one-way ANOVA with Tukey’s multiple comparisons test. Data in S1 Data.

Since T2.KD decreased SynT aggregation, we evaluated how it affected SynT clearance in the cell. For this purpose, we performed a time-chase experiment blocking de novo protein synthesis with cycloheximide (CHX). We did not observe significant differences in the levels of SynT between T2.KD or Scr cells (Fig 4A). Since the autophagy lysosome pathway (ALP) is described as the main mechanism to clear high-molecular-weight species of aSyn that cannot be degraded by the proteasome [17], we evaluated the effects of T2.KD on autophagy. Remarkably, although we observed no differences in the ALP in naïve cells, T2.KD significantly enhanced the activity of the ALP in the SynT aggregation model, as seen by the increased accumulation of LC3-II levels after 2 h of ALP inhibition with bafilomycin A (Fig 4B). To further evaluate these findings, we scored the number of LC3 punctae in T2.KD and Scr cells in both naïve cells or in the SynT aggregation model using immunocytochemistry for LC3 (Fig 4C). In agreement with the previous data, LC3 punctae were not modulated by T2.KD in naïve cells. However, in the aSyn aggregation model, T2.KD increased the number of LC3 punctae per cell (Fig 4C). Consistently, upon T2.KD, we observe a basal decrease in the levels of SQSTM1 (P62) in the cell model of aSyn aggregation (S4 Fig). In total, these results suggest that while basal autophagy is not affected by SIRT2, upon aSyn aggregation, SIRT2 inhibition potentiates the activity of ALP, thereby efficiently clearing aSyn aggregates.

### aSyn acetylation is neuroprotective in primary cortical neurons

**Fig 5:**
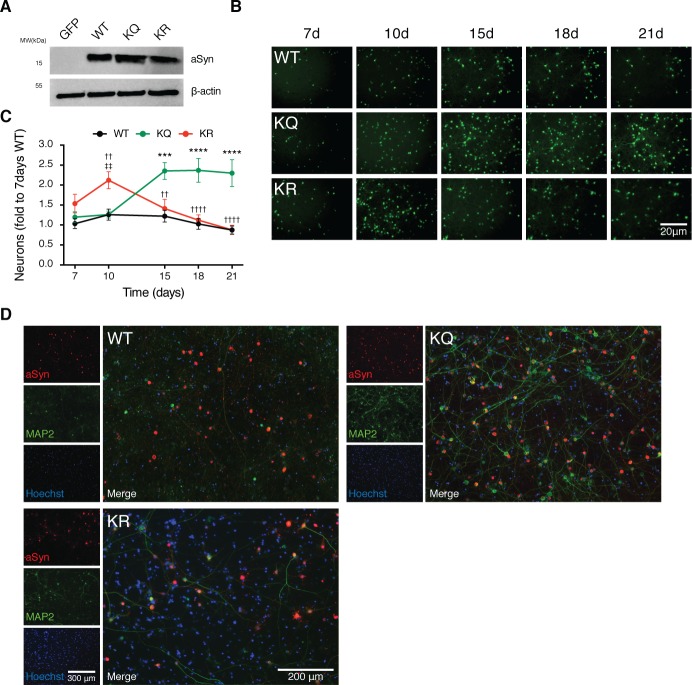
aSyn acetylation mimic is neuroprotective in primary cortical neurons. **(A)** Cultured primary neurons were transduced with AAVs encoding for EGFP, WT aSyn, KQ, or KR-mutant aSyn at day in vitro 3 (DIV3). Whole-cell lysates were analyzed by immunoblotting 7 d postinfection with antibodies against aSyn and β-actin (*n* = 3). **(B)** Primary neuronal cells were coinfected with aSyn and EGFP at DIV3 and monitored over time. 16 images per condition were acquired, and the EGFP fluorescence signal was recorded in living neurons at 7, 10, 15, 18, and 21 d posttransduction (*n* = 3). Representative images are shown. Scale bar 20 μm. **(C)** Total number of EGFP positive cells normalized to the number of neurons on WT 7 d posttransduction is presented (*n* = 6). Data in all panels are average ± SD, * *p* < 0.05, ** *p* < 0.01, *** *p* < 0.001, two-way ANOVA with Bonferroni post-test. **(D)** Cortical neuronal cells 15 d posttransduction were processed for ICC (aSyn, red; microtubule-associated protein 2 [MAP2], green; nuclei, Hoechst, blue). Representative images are presented. Scale bar 200 μm. Data in S1 Data.

As we showed that SIRT2 regulates aSyn acetylation and that reduction of SIRT2 levels is protective in a cell model of PD, we next aimed at validating our findings in rat primary cortical neuronal cells. It was previously described that 10 d posttransduction of primary cortical neurons with adeno-associated viruses (AAV) encoding for WT aSyn results in severe neuronal loss [18]. Based on this model, rat cortical neurons were transduced with AAV6 vectors encoding WT, KR, or KQ aSyn under the synapsin promoter for neuronal expression (S5 Fig). We detected similar expression levels of WT and of the two aSyn acetylation mutants 7 d post-viral transduction (Fig 5A). Remarkably, we observed that cells expressing the acetylation-mimic mutant (aSyn KQ) displayed a significant reduction in the toxicity observed 10 d posttransduction (31%) (S6 Fig). However, two to three weeks after transduction, no significant differences were detected (S6 Fig). Next, we performed live cell imaging to determine the effect of aSyn intrinsic acetylation in the number of living neurons over time. Neurons were cotransduced with aSyn (WT or mutant) and enhanced GFP (EGFP), and EGFP-positive neurons were counted at different time points (Fig 5B and 5C). At 7 d posttransduction, the number of transduced neurons was identical for all conditions. Between 15 and 21 d posttransduction, both WT and KR aSyn induced pronounced neuronal loss, while the number of living neurons in cultures expressing the acetylation mimic mutant KQ was significantly higher (2-fold) (Fig 5B and 5C). We next examined the distribution and intracellular localization of aSyn in neurons 15 d posttransduction. Using an antibody that specifically recognizes human aSyn, we did not observe major differences on the subcellular distribution of the different aSyn variants—all were distributed throughout the neurons, both in cell bodies and neurites (Fig 5D). Strikingly, both the WT and the KR aSyn severely affected the dendritic phenotype, promoting an evident shortening and loss of dendritic arborisation (Fig 5D). In contrast, the dendritic structure was preserved in neurons expressing the KQ mutant, as shown by microtubule-associated protein 2 (MAP2) staining (Fig 5D). Overall, both WT and KR—but not KQ aSyn—promoted prominent neurodegeneration and loss of dendritic arborisation, suggesting aSyn acetylation is protective.

### Blocking aSyn acetylation induces nigral dopaminergic neuronal loss

In order to investigate the effect of aSyn acetylation in vivo, we stereotaxically injected AAV6 viruses driving the neuronal expression of WT, KR, or KQ mutant aSyn into the rat substantia nigra (SN). First, we established the neurotoxicity of AAV6-mediated expression of WT aSyn at three different time points (S5A and S5B Fig). We observed that 6 d after injection of WT aSyn, no significant tyrosine hydroxylase (TH) loss was detected. However, 12 d postinjection, the number of TH-positive cells was significantly reduced in comparison to that observed in the GFP-injected group. No additional TH-cell loss was observed 18 d postinjection, indicating the lesion did not increase between 12 and 18 d postinjection. As expected, the expression of GFP did not induce TH-positive neuronal loss (S7A and S7B Fig).

**Fig 6:**
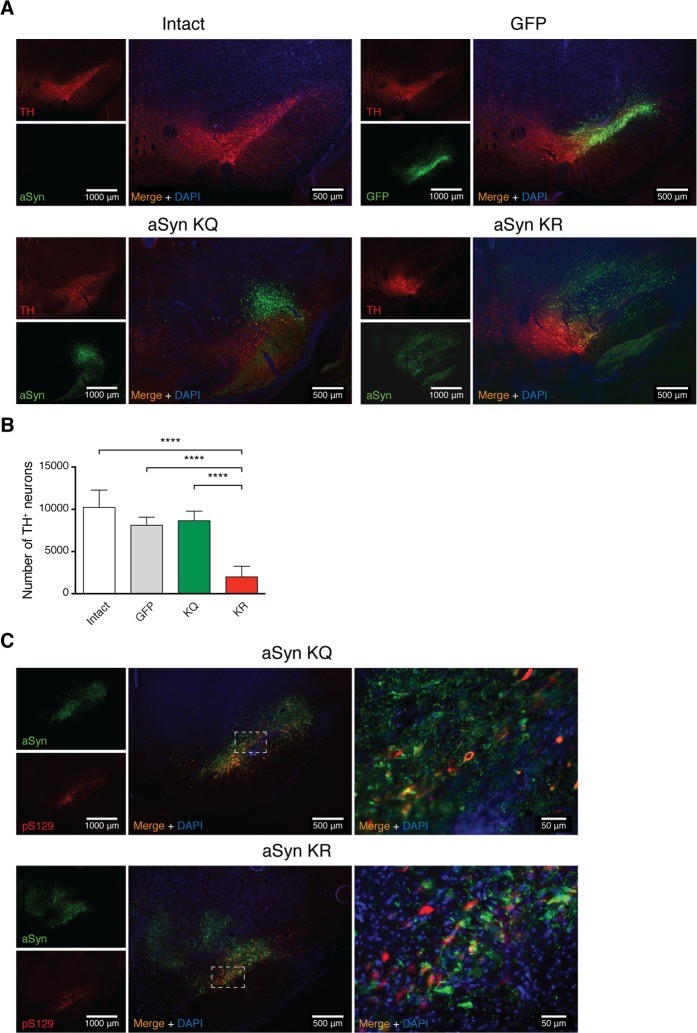
aSyn acetylation-resistant mutant induces nigral dopaminergic neuronal loss in vivo. **(A)** AAV6-mediated delivery of EGFP and mutant aSyn (KQ or KR) into the SN of the rat brain. TH and GFP or aSyn expression was examined in brain sections 3 wk after injection by immunohistochemistry (TH, red; GFP or aSyn, green; DAPI, blue). Representative sections are shown. Scale bar for isolated channels 1,000 μm and for merged channels 500 μm. **(B)** Stereological counting of the number of TH-positive neurons in the SN. The contralateral SN of the different groups of animals was used as a control (intact). Data in panels are average ± SD. **(C)** Brain sections stained for aSyn (green), pS129 aSyn (red), and DAPI (Blue). Representative sections are shown. Dashed square boxes delineate the magnification presented on the right. Scale bar for isolated channels 1,000 μm and for merged channels 500 μm and 50 μm. *** *p* < 0.001, **** *p* < 0.0001, one-way ANOVA with Bonferroni correction used for statistical calculations. In (B), GFP was used as a control; *n* = 6–7 animals per condition; five sections from a one-in-six series were analyzed per brain. Data in S1 Data.

Next, we compared the neurotoxicity of the KQ and KR aSyn mutants using the same paradigm. Based on the data obtained for WT aSyn, we assessed the effect 3 wk postinjection. Stereological quantification of TH-positive cells revealed a significant loss of TH-positive neurons 3 wk post-KR aSyn expression (Fig 6A and 6B). In agreement with the in vitro results, the TH-positive cell loss in cells expressing the KQ aSyn mutant was identical to that observed in control conditions (GFP expression) (Fig 6B). On the other hand, the KR mutant induced strong loss of TH-positive cells in the SN.

We next investigated whether interfering with aSyn acetylation on K6 and K10 affected aSyn pathology in vivo. The expression of both KQ and KR aSyn variants in the SN resulted in the detection of phosphorylated aSyn on Ser129 (pS129) (Fig 6C), suggesting the accumulation of pathological forms of aSyn. However, the subcellular localization of the pS129 signal was different depending on the aSyn variant. While the KQ mutant displayed a predominantly cytoplasmic staining, the KR mutant displayed a homogeneous pS129 staining throughout the cells (Fig 6C). We also assessed aSyn pathology by immunostaining with the 5G4 antibody, which reacts with aggregated forms of aSyn [19] (S8 Fig). As expected, all variants tested resulted in immunostaining with this antibody, suggesting they all formed aSyn aggregates. Unexpectedly, we observed a stronger staining in rats expressing WT aSyn when compared to that observed in animals expressing the acetylation mutants (S8A Fig). However, as observed for the pS129 staining, immunostaining with the 5G4 antibody also revealed different aSyn subcellular localization depending on the mutant expressed. The KQ mutant appeared predominantly in the cytoplasm, being excluded from the nucleus in most cells. In contrast, the signal for both KR mutant or WT aSyn was homogeneously distributed throughout the cells (S8B Fig).

### SIRT2 deletion protects from aSyn or MPTP toxicity in vivo

**Fig 7:**
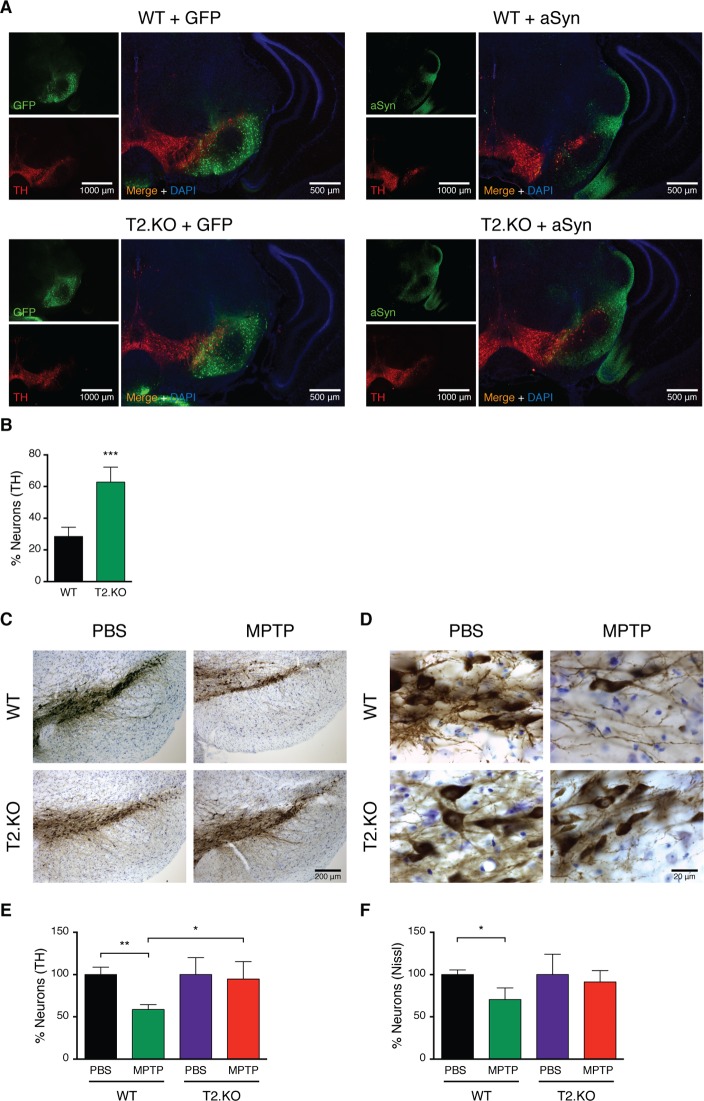
Knockout of SIRT2 protects from aSyn or MPTP toxicity in mice. **(A)** AAV6-mediated delivery of GFP or WT aSyn into the SN of WT or T2.KO mice brains. TH and GFP or aSyn expression was examined in brain sections 2 wk postinjection by immunohistochemistry (TH, red; GFP or aSyn, green; DAPI, blue). Representative sections are shown. Scale bar for isolated channels 1,000 μm and for merged channels 500 μm. **(B)** Stereological counting of the number of TH-positive neurons in the SN. The number of TH-positive neurons in the SN of GFP-injected mice was used as control (at least *n* = 3 per group). Data, presented as percentage of TH-neurons, are average ± SD. *** *p* < 0.001, unpaired *t* test with equal SD. Chronic MPTP treatment of WT or T2.KO mice brain. TH **(C)** or neurons (Nissl) **(D)** were examined in brain sections 2 wk postinjection by immunohistochemistry (TH, DAB; Neurons, Nissl; DAPI, blue). Representative sections are shown. Scale bar 200 μm. Stereological counting of the number of TH-positive **(E)** or Nissl-positive neurons **(F)** in the SN (at least *n* = 4 per group). Neuron numbers in NaCl-injected mice were used as normalization controls, and data are expressed as percentage of the corresponding NaCl-injected control animals. For (E) and (F), data (presented as percentage of neurons), are average ± SD. * *p* < 0.05, ** *p* < 0.01, two-way ANOVA followed by Tukey’s multiple comparisons test. Data in S1 Data.

We showed that the levels of acetylated aSyn were increased in T2.KO mice (Fig 1E and 1F). Next, to assess the effect of SIRT2 on aSyn-mediated neurodegeneration in vivo, we evaluated whether deletion of SIRT2 protected against TH-cell loss. We stereotaxically injected AAV6 virus driving the neuronal expression of WT aSyn into the SN of WT or T2.KO mice. 2 wk after viral injection, TH neuronal loss was observed in WT mice (28.5 ± 2.4% surviving neurons). Remarkably, in T2.KO mice, we observed a clear protection (62.8 ± 5.5% surviving neurons) (Fig 7A and 7B).

We further investigated the protective effects of SIRT2 deletion in the chronic MPTP mouse model of parkinsonism [20]. As expected, 2 wk post-MPTP injection, we observed a significant decrease in the percentage of TH neurons in WT mice (Fig 7C–7E). Overall neuronal loss was also observed (Fig 7F). In contrast, we observed no neuronal loss in T2.KO animals (neither TH or overall neurons) (Fig 7C–7F).

## Discussion

Aging is, by far, the major risk factor for PD and other neurodegenerative disorders, but the precise molecular mechanisms involved are still elusive. The sirtuin family of deacetylases regulates many aspects of the aging process. Intriguingly, several sirtuins have emerged as putative therapeutic targets in several neurodegenerative diseases. For example, SIRT1 overexpression has proven beneficial in models of Alzheimer disease, Huntington disease, and PD [1,21]. However, while SIRT1 is ubiquitously expressed in the body, SIRT2 is the most abundant sirtuin in the brain and is primarily present in the cytoplasm of brain cells (neurons and oligodendrocytes). The levels of SIRT2 increase with aging [3], and we previously showed that pharmacological inhibition of this sirtuin is protective in cellular, *Drosophila*, and mouse models of PD but not in models of amyotrophic lateral sclerosis [4,22]. Nevertheless, the precise mechanisms of SIRT2-mediated protection against aSyn aggregation and toxicity are still unclear.

In the present study, we provide strong evidence demonstrating that SIRT2 inhibition robustly alleviates neuropathology in several models of synucleinopathy by regulating the levels of aSyn acetylation. We show, for the first time, that SIRT2 interacts with and deacetylates aSyn in the highly conserved N-terminal domain.

We started with the premise that acetylation balance is impaired in neurodegeneration. Thus, we first hypothesized that SIRT2-dependent modulation of aSyn acetylation could be the basis for aSyn pathogenicity. It was previously shown that aSyn is acetylated in the N-terminus [8,23] and that this modification is important for the interaction of aSyn with lipids, as acetylation increases membrane-binding properties of aSyn by increasing its alpha helical content and reducing its ability to aggregate [24]. Other posttranslational modifications are also known to affect the structure, membrane-binding ability, and oligomerization properties of aSyn [25]. Since aSyn is a very lysine-rich protein, with 15 putative acetylation sites located mainly in the N-terminal region of the protein [26], it was reasonable to hypothesize that aSyn could be naturally acetylated in lysine residues in addition to the N-terminal acetylation and that these residues could, perhaps, be substrates for SIRT2 deacetylation. In our study, and in agreement with previous findings, we confirmed that aSyn is acetylated in the N-terminus. However, we also detected acetylation on residues K6 and K10 (Fig 1A and S1 Table). Importantly, a previous proteomic analysis of lysine acetylation in rat tissue also detected aSyn acetylation on K6 [27]. Here, we also demonstrated that SIRT2 interacts with and deacetylates aSyn.

We previously reported that pharmacological inhibition of SIRT2 reduces aSyn toxicity and decreases aggregation [4]. Therefore, to exclude potential off-target effects of SIRT2 inhibitors, we evaluated the effects of genetic inhibition of SIRT2 on aSyn aggregation and toxicity in a well-established cellular model of aSyn aggregation. Strikingly, we found that by elevating the levels of aSyn acetylation, knockdown of SIRT2 was able to reduce aSyn aggregation and toxicity. Therefore, we reasoned that aSyn acetylation might be protective. To evaluate the intrinsic implications of aSyn acetylation on K6 and K10, we generated acetylation-mimic (KQ) and acetylation-resistant (KR) mutants. Using the cell-based model of aSyn aggregation, we confirmed that while the majority of cells expressing the KR mutant displayed aSyn inclusions, those expressing the KQ displayed almost no inclusions. Moreover, we showed that recombinant aSyn acetylation mutants display the same trend. Notably, combining WT aSyn with the KQ acetylation-mimic aSyn suppressed fibrillisation, while combining WT with the KR acetylation-resistant mutant potentiated aSyn fibrillisation.

The fact that KQ aSyn is able to suppress WT aSyn fibrillisation suggests that, in normal conditions, the acetylated form of aSyn might be dominant. This observation raises the important and provocative question of how much acetylated aSyn is necessary in the brain in order to prevent fibrillisation as we age. Going one step further, this might enable us to predict what are the chances of developing PD later in life by measuring the levels of SIRT2 and/or acetylated aSyn and could shed light into the question of why some people end up developing PD later in life.

Both NMR and far UV-CD data showed that the presence of acetylation-resistant modifications do not affect the binding of aSyn to lipid vesicles (S3A Fig). In contrast, mimicking aSyn acetylation reduces the ability of the N-terminal region to bind membranes, preventing this region from acting as a membrane-attachment anchor (Fig 3D and 3E). Importantly, this finding suggests that acetylation may regulate the physiological role of aSyn on vesicular trafficking [28,29] and should be further investigated in future studies.

Protein acetylation is known to modulate the clearance of different proteins. For example, acetylation of huntingtin—another intrinsically disordered and misfolding-prone protein—on K444 facilitates its clearance via the ALP [30]. In addition, SIRT2 has been previously associated with autophagy. First, when SIRT2 releases FOXO1, the latter gets acetylated and binds to ATG7, inducing autophagy in the context of cancer [31,32]. Second, overexpression of SIRT2 inhibits autophagic turnover by interfering with aggresome formation [33]. Therefore, we asked whether SIRT2 could also modulate the clearance of aSyn, mainly by interfering with the ALP. Although we did not observe significant differences in the rate of aSyn clearance, we found that T2.KD induced macroautophagy only in the presence of aSyn inclusions (Fig 4B and 4C and S4 Fig). Since we observed that acetylated aSyn is less prone to aggregate and could even prevent the aggregation of the WT form and that T2.KD promoted a more efficient clearance of the aggregates, we propose that SIRT2 acts on several fronts, potentiating neurodegeneration. On one side, SIRT2 affects the conformation of aSyn through deacetylation, thus rendering it more prone to aggregate. Simultaneously, it regulates the main clearance pathway for aggregated aSyn. Therefore, we propose a model in which the age-dependent increase of SIRT2 in the brain, with the concomitant decrease of acetylated aSyn, comes with two major deleterious consequences: an increase of aggregation and the exacerbation of the expected defects in the ALP associated with aging [34].

We then assessed the effects of aSyn acetylation in rat primary cortical cultures. By analysing the expression of aSyn acetylation mutants over time, we observed that while expression of the acetylation-resistant mutant aSyn induced a similar reduction in the number of neurons to that observed for WT aSyn, the acetylation-mimic mutant was less toxic. Expression of the same aSyn variants in vivo in the rat SN confirmed that the acetylation-mimic KQ mutant was less toxic to TH-positive neurons that the KR mutant or than WT aSyn. Interestingly, although the immunoreactivity for pS129 or aggregated forms of aSyn was identical for both the KQ and KR mutants, the intracellular distribution of the protein was altered. Altogether, this suggests that acetylation may not completely abolish the ability of aSyn to aggregate in vivo but that it may also modulate the subcellular distribution of the protein, thereby modulating its toxicity.

Given the protective effects of mimicking aSyn acetylation in vivo, we also evaluated the putative protective effects of SIRT2 deletion. For this we used two in vivo models reporting on different aspects of PD: aSyn toxicity and TH-cell loss. Importantly, we found that SIRT2 deletion was protective in both the AAV-mediated model of aSyn expression in the SN and in the chronic MPTP model of parkinsonism.

In summary, our data suggest that acetylation of aSyn on K6 and K10 may regulate the distribution (and perhaps the function) of aSyn, also modulating its aggregation potential and toxicity. We identify acetylation as a mechanism for removing aSyn by targeting it to autophagy. Thus, SIRT2 activity is an important mediator of aSyn acetylation, underlying its potential as a target for therapeutic intervention in synucleinopathies and, possibly, in other neurodegenerative disorders in which protein acetylation may play underappreciated roles in modulating protein homeostasis.

## Materials and methods

### Ethics statement

This study does not involve human participants or tissue. All experimental animal procedures were conducted according to approved experimental animal licenses, issued by the responsible animal welfare authority (Niedersächsisches Landesamt für Verbraucherschutz und Lebensmittelsicherheit) and controlled by the local animal welfare committee and veterinarians at the University Medical Center Göttingen after approval from the ethics committee.

### Mass spectrometry analysis

Total protein extracts from C57Bl/6 mice brain samples were processed as in [7]. Mass spectrometry was performed on an Applied Biosystems 4700 Proteomics Analyzer with TOF/TOF ion optics as previously described [7]. All peptide mass values were considered monoisotopic, and a MS mass tolerance was set at 100 ppm. Trypsin was assigned as digestion enzyme of aSyn. A triple miss cleavage was allowed and oxidation of methionyl residues, acetylation of the N-terminus, and acetylation of lysine residues were assumed as variable modifications. All peaks with S/N greater than 5 were included for matching against in silico digestion of corresponding aSyn sequence (*Mus muscullus*) in mMass software [35].

### H4 and HEK 293T cell culture and transfection

Cells were cultured at 37°C in a humidified incubator containing 5% CO2 (g). H4 cells were grown in Opti-MEM medium (Gibco-Invitrogen), and HEK 293T cells were grown in DMEM with GlutaMax medium (Gibco-Invitrogen); both were supplemented with fetal bovine serum (FBS) (10% v/v). Cells were transfected with FuGENE 6 transfection Reagent (Roche) as previously described [7]. Briefly, 293T cells were transfected with GFP or SIRT2 fused with GFP (SIRT2::GFP) or cotransfected with GFP with aSyn or SIRT2::GFP with aSyn.

### Immunoprecipitation

Protein immunoprecipitation was performed as we previously described [36].

### Immunoblotting

Cells lysis and immunoblotting was performed as previously described [7]. For immunoblotting, we used the following antibodies: anti-aSyn (BD Transduction laboratories, S63320, 1:3000), anti-V5 (Santa Cruz, SC-83849-R, 1:1000), anti-LC3 (Nano Tools, 0260-100/LC3-2G6, 1:2000), anti–β-actin (Ambion, AM4302, 1:5000), anti-GAPDH (Ambion, AM4300, 1:5000), anti-GFP (NeuroMab, P42212, 1:3000), and anti–acetyl-lysine (Cell Signaling, 9441S, 1:1000).

### Plasmids for aSyn recombinant protein expression

aSyn double mutants were generated by site-directed mutagenesis to mimic the acetylated or the nonacetylated forms of aSyn, replacing the lysine on positions 6 and 10 by glutamine (K6 + 10Q = KQ) or arginine (K6 + 10R = KR), respectively.

### Acetylation studies in mice

Animal experiments were performed according to institutional and national regulations. All mice used were in congenic C57Bl/6. SIRT2 knockout mice have been described previously [37]. All mice were housed at controlled temperature (25°C) and 12:12 h light/dark cycle. For protein analysis, brains from mice at the different ages (2 and 8 mo old) were quickly removed, striata were dissected, and samples were homogenized in RIPA buffer in the presence of protease and phosphatase inhibitors (Roche Complete and PhosStop). Samples were then rotated for 1 h at 4°C and centrifuged at 18,000 g for 30 min. For further analyses, we used the soluble fractions. aSyn was partially purified and enriched as described in [7]. For immunoblotting, we used anti aSyn and antiacetylated lysine antibodies.

### Recombinant Sirt2 production

Recombinant SIRT2 (43–356) was expressed in *E*. *coli* and purified through affinity and size-exclusion chromatography as previously described in [38].

### Chemical acetylation and enzymatic deacetylation

The buffer of aSyn was exchanged to 100 mM sodium phosphate pH 7.4 using a NAP-5 column (GE Healthcare). aSyn was subsequently treated with acetic anhydride, 13 mM final concentration (10-fold molar excess compared to the lysine concentration), by four consecutive, stepwise additions over an hour followed by an extra hour of incubation on ice. Excess acetic anhydride was then quenched by addition of 20 mM Tris pH 8.0.

For the enzymatic deacetylation, 1.1 μM chemically acetylated aSyn was incubated for 1 h on ice in the presence or absence of 0.11 μM SIRT2 and 1 mM NAD^+^, and intact protein masses were then determined through HPLC-coupled ESI-MS. Proteins were concentrated and washed on a Piccolo Proto 200 C4 5μm 2.5 x 0.5mm trap column (Higgins Analytical, Mountain View, California) and subsequently switched in line with and separated on a Jupiter C4 5μm 300Å 150 x 1 mm analytical column (Phenomenex, Torrance, California) mounted onto a Shimadzu Prominence UFLC (Shimadzu, Duisburg, Germany) at a 70μl min^-1^ flow rate with the following buffers: A—5% ACN, 5% DMSO, and 0.1% FA; B—90% ACN, 5% DMSO, and 0.1% FA. Proteins were then eluted over with a gradient of 3 min of 1% B to 55% B followed by 1 min of 55% B to 90% B. Mass analysis was performed by ESI-TOF-MS on an AB Sciex TripleTOF 5600+ mass spectrometer (Sciex, Darmstadt, Germany) with a DuoSpray Ion Source with the following settings: floating voltage of 5,500 V, temperature of 350°C, and declustering potential of 120 with 4 separate TOF experiments each, respectively, with 4, 12, 20, and 40 time bins summed. Spectra were integrated over a retention time period, and the summed TOF experiment with the greatest resolution was selected. The raw data was then converted and deconvoluted using the MaxEnt I algorithm (Waters, Milford, Massachusetts) at a resolution of 0.1 Da.

### In vitro deacetylation of semisynthetic acK6 and acK10

Chemical acetylated aSyn at lysine 6 (acK6) or 10 (acK10) was obtained by using the semisynthetic approach [9,10]. acK6 or acK10 was incubated in the presence or absence of both recombinant SIRT2 in increasing amounts and NAD^+^ (1 mM) at 37°C in SDAC buffer (50 mM Tris·HCl [pH 9.0], 4 mM MgCl_2_, 50 mM NaCl, 0.5 mM DTT). 3 h postincubation, the reaction was stopped by the addition of protein sample buffer.

### Viral production and infections of SIRT2 shRNA

HEK 293T cells (1.5 x 10⁶) were cotransfected with Δ8.9, VSVG, and SIRT2 shRNA plasmids (1.8μg, 0.2μg, and 2μg, respectively—Sigma RNAi facility) using FuGENE6 (Promega, Wisconsin, United States). 48 h posttransfection, the lentiviruses were collected and filtered (0.45μm). H4 cells (1 x 10^5^) were infected with the lentivirus supplemented with PolyBrene (10μg/mL) (Sigma). After 4 h, medium was replaced by Opti-MEM supplemented with FBS. 48 h postinfection, cells were selected using Puromycin-Dihydrochloride (1μg/mL; Sigma).

### aSyn aggregation model of human cells

The aggregation model of H4 neuroglioma cells was used as previously [36]. Immunocytochemistry was performed as previously [36].

### Sucrose gradient

Total protein from Scr or T2.KD H4 cells transiently expressing SynT with synphilin-1 was processed on a 5% to 30% sucrose gradient as previously described [39].

### Triton insolubility assay

Total protein (500 μg) from Scr or T2.KD cells transiently expressing SynT and synphilin-1 for 48 h was incubated with 1% Triton X-100 on ice for 30 min. Protein fractions were separated by centrifugation at 15,000 g for 60 min at 4°C. Soluble protein fraction was collected and the insoluble protein fraction pellet was resuspended in 40 μL of 2% SDS Tris-HCl buffer pH 7.4 and sonicated for 10 s. Total protein and T-Insoluble fractions (5 μl of each) were loaded and resolved by SDS-PAGE and immunoblotted as previously described.

### LDH cytotoxicity assays in H4 cells

Cytotoxicity was measured using LDH kit (Clontech) as previously described [14].

### Recombinant aSyn aggregation assay

The expression and purification of human aSyn was performed as previously described [7]. aSyn aggregation assay was performed as previously described [14]. WT and acetylation mutants K6+10Q or K6+10R were combined in a 1:1 ratio.

### NMR spectroscopy

^1^H-^15^N HSQC spectra of ^15^N-labelled wild-type and acetylation mutants K6+10Q or K6+10R aSyn were acquired at 15°C on a 600 MHz Bruker spectrometer in HEPES 25 mM, pH 7.0, NaCl 50 mM, 10% D_2_O. aSyn backbone resonance assignment was transferred from previous studies [40]. In case of K6+10Q or K6+10R aSyn, peak centers were adjusted only in regions of small signal overlap. Peaks affected by severe overlap were excluded from the analysis. NMR data were processed and analyzed by using NMRPipe [41] and Sparky (T. D. Goddard and D. G. Kneller, University of California, San Francisco).

For NMR and CD measurements (see below) of aSyn in the presence of SUVs, SUVs were prepared with 1-palmitoyl-2-oleoyl-sn-glycero-3-phosphocholine (POPC) and 1-palmitoyl-2-oleoyl-sn-glycero-3-phosphate (POPA) (Avanti Polar Lipids) at a 1:1 molar ratio using previously published protocols [42].

### Circular dichroism

Far UV-CD measurements were performed on a Chirascan (Applied Photophysics, United Kingdom) circular dichroism spectrometer using protein concentrations of 10 μM in HEPES buffer, 10 mM NaCl, pH 7.4, in a quartz cuvette with a 0.1 cm light-path. To probe the binding of aSyn to SUVs, increasing concentrations of SUVs were added (from 1:20 to 1:270 protein-to-lipid molar ratio). Each experiment was repeated at least twice. Baseline correction was performed with the same buffer. Data were expressed as mean residue ellipticity (degree cm^2^ dmol ^–1^). Affinity curves were fitted with a single exponential equation considering a one site–specific binding model.

### aSyn clearance

For CHX chase experiments, Scr or T2.KD H4 cells were transfected with SynT as previously [7]. 40 h posttransfection, cells were treated with cycloheximide (100 μM, added at given time points). Protein extracts were immunoblotted. Autophagy activity studies were performed as previously described [43]. Activity was given by the amount of accumulated LC3 after 2 h of treatment with the autophagy blocker bafilomycin A. Basal levels of SQSTM1 (p62) were also measured (sc-28359, Santa Cruz Biotechnology). For LC3 punctae analysis, 1.2 x 10^5^ cells were seeded on MatTek dishes. 48 h after plating, cells were carefully washed three times with PBS and processed for immunocytochemistry, as previously described [36], using an anti-LC3 antibody. Fluorescence was detected by the use of a widefield fluorescence microscope or a confocal microscope. The number of LC3 punctae per cell was determined.

### Generation of plasmids for the production of adeno-associated viruses (AAV)

For the generation of the AAV vectors of serotype 6 (AAV-6) constructs encoding for human WT aSyn and lysine mutants (K6+10Q and K6+10R), cDNA was amplified by PCR using the following primers: Forward Primer sequence for WT aSyn: 5′- GGCAGATCTACCGGTCGCCACCATGGATGTATTCATGAAAGGACTTTCAA AGGCCAAGGAGGG -3′; Forward Primer sequence for K6+10Q aSyn mutant: 5′- GGCAGATCTACC GGTCGCCACCATGGATGTATTCATGCAGGGACTTTCACAGGCCAAGGAGGGA -3′; Forward Primer sequence for K6+10R aSyn mutant: 5′- GGCAGATCTACCGGTCGCCACCATGGATGTATTCATGAGAG GACTTTCAAGGGCCAAGGAGGGA -3′; Reverse Primer sequence for aSyn WT sequence: 5′-CCCGC GGCCGCTTAGGCTTCAGGTTCGTAGTCTT -3′. PCR products were digested with restriction enzymes and cloned into pAAV-*6P*-*SEWB*.

### AAV vector preparation

WT and mutated aSyn was expressed from human synapsin-1 (hSyn1) gene promoters, followed by WPRE and bovine growth hormone polyadenylation site according to standard procedures (S2 Fig) [44]. Briefly, recombinant AAV-6 for expression of WT, KQ (K6+10Q), or KR (K6+10R) were produced by transient transfection in HEK293 cells, purified by iodixanol gradient ultracentrifugation and heparin affinity chromatography, and dialysed against PBS. Vector titres were determined by qPCR, and purity of viral particles was confirmed to be >98% by SDS-PAGE. Vector titres were 5 x 10e12 vg/ml for WT and K6/10R synuclein and 8 x 10e12 vg/ml for K6/10Q synuclein.

### Primary cell culture

Primary cortical cultures were prepared from Wistar rats at embryonic day 18, as previously described [45]. Cortical cells were seeded in poly-ornithine (PLO) precoated glass coverslips (13 mm) at a density of 250,000 cells/well, and were maintained in Neurobasal medium (Gibco) supplemented with 1% Penicillin-Streptomycin (Pan-Biotech), 0.5 mg/mL Transferrin (Sigma), 125mM L-glutamine (Pan-Biotech), and 1 x B27 (Gibco). Cultures were grown at 37°C in humidified 5% CO_2_ atmosphere. At DIV3, neuronal cells were infected with equimolar amounts of recombinant adeno-associated virus (AAV-6), under the synapsin promoter, encoding for EGFP, WT, or mutant aSyn (1 x 10^8^ vg/250,000 neurons/well). For live cell imaging experiments, cells were cotransduced with 1 x 10^4^ vg EGFP/250,000 neurons/well.

### Live cell imaging

Transduced cells were imaged on an Olympus IX81-ZDC microscope system (Olympus) with a 10x objective. An EGFP fluorescence signal was recorded with constant exposure time (300 ms) from living neurons at 7, 10, 15, 18, and 21 d posttransduction. A total of 16 images per well were randomly and automatically collected for quantification analysis. The total number of GFP-positive cells was counted for each condition using the Olympus Scan R Image Analysis Software and normalized to EGFP + WT aSyn.

### Primary cultures toxicity measurements

Supernatants were collected from primary cells on 10, 15, and 18 d posttransduction. Cell viability was assessed by quantitatively measuring LDH as previously mentioned. For background control, noninfected cultures were used, and maximal LDH release was measured in the supernatants of primary cells lysed with 2% Triton X-100. Percentage of toxicity was calculated as indicated by the manufacturer.

### Cortical neurons immunohistochemistry

Cortical primary cells growing on glass coverslips were fixed for 10 min with 4% paraformaldehyde 15 d after transduction. Following three washes with PBS, cells were permeabilized for 15 min with 0.5% Triton/PBS and blocked with 3% BSA/PBS for 1 h at room temperature (RT). Human anti-aSyn antibody (MJFR1, abcam #ab138501) and anti-MAP2 (Sigma #M9942) were prepared 1:1,000 in blocking solution and incubated overnight, at 4°C. Coverslips were carefully washed three times with PBS and incubated with secondary Alexa Fluor antibodies (Life Technologies) diluted 1:1,000 in blocking buffer at RT. Nuclei were stained with Hoechst, and after three washes with PBS, coverslips were embedded in moviol. Imaging was performed using an epifluorescence microscope (Leica DMI 6000B microscope, Leica).

### Stereotaxic injections in rats

Young adult female Wistar rats (230–260g each; Janvier, Saint Berthevin, France) were used for AAV injections. Rats were housed in a temperature-controlled room that was maintained on a 12-h light and/or dark cycle. Food and water were provided ad libitum.

### Stereotaxic injections in the rat SN

All surgical procedures, intracerebral stereotaxic vector injections into the right hemisphere SNpc (coordinates were as follows: AP: –4.7; ML: –2.2; DV: –7.6 mm relative to Bregma, tooth bar: –3.3 mm) (S3C Fig), and tissue preparations were performed essentially as described [18].

In a first attempt, young adult female Wistar rats (230–260g) were injected with 2 μL of control GFP or human WT aSyn AAV6 virus (~1x10^8^ vector genomes [vg]/ml) into the right SN at a flow rate of 0.2 μl/min. Animals were PFA-perfused 1, 2, and 3 wk after virus injection.

In a second attempt, young adult female Wistar rats (230–260g) were injected with 2 μL of control GFP, human WT, or mutants KR and KR aSyn AAV6 virus (~1x10^8^ vg/ml) into the right SN at a flow rate of 0.2 μl/min. Animals were PFA-perfused 3 wk after virus injection. 40-μm thick serial coronal sections from the SN were collected on a cryostat (Leica) and processed for immunohistochemistry stainings.

### Immunohistochemistry of rat brain sections

#### Rat tissue preparation

Rats were transcardially perfused with 150 ml in phosphate buffer (PB) followed by 150 ml 4% PFA in PB at a flow rate of 15 ml/min. The brains of the animals were removed, postfixed overnight in the same solution, then cryoprotected by immersion in 30% sucrose for 36 h. Free-floating 40 μm serial coronal sections from throughout the SN were collected with a Cryostat (Leica Microsystems, Germany). Brain slices were placed in a storage solution (30% glycerol, 30% ethylene glycol in 0.1 M PB) and stored at –20°C before use.

#### Fluorescent stainings

For the detection of fluorescent proteins after infection, free-floating sections were washed three times in phosphate-buffered saline (PBS), blocked in PBS containing 4.5% bovine saline albumin (BSA) for 1 h, then incubated overnight at 4°C in PBS containing 3% BSA, 0.2% Triton X-100, and one of the following antibodies of interest. Sections were rinsed three times in PBS and incubated with Alexa Fluor 488-labeled antimouse or rabbit IgG or Alexa Fluor 555-labeled antimouse or rabbit IgG (Invitrogen) at room temperature for 1 h. The sections were rinsed in PBS and the nuclei were counterstained with DAPI (dilution 1:10,000, Wako) for 3 min. The sections were mounted in Mowiol.

Series of sections were stained for aSyn to localize the injection site in aSyn-injected rats. Tyrosine hydroxylase (TH) (Rabbit, Millipore, ab152) immunostaining was used in order to quantify the loss of dopaminergic neurons in the injected rats. The following antibodies were used to detect specific synucleins: N-terminal rat aSyn (Mouse, Bd Transduction Laboratories, 610787), recognized amino acid 121–125 of human aSyn (Mouse, Santa-Cruz, SC-12767), pS129 aSyn (Rabbit, Abcam, ab51253), and aggregated forms of aSyn (Mouse, Millipore, clone 5G4, MABN389).

### Stereotaxic injections in the SN

Mice were housed in a temperature-controlled room that was maintained on a 12 h light and/or dark cycle. Food and water were available ad libitum. Both female and male WT and SIRT2-KO mice were used for AAV injections. All surgical procedures, intracerebral stereotaxic vector injections into the right hemisphere SNpc (coordinates were as follows: AP: –3.2; ML: –1.2; DV: –4.4 mm relative to Bregma, tooth bar: 0.0 mm), and tissue preparations were performed essentially as described [46]. Mice were injected with 2 μL of control GFP or human WT aSyn AAV6 virus (~1x10^8^ vg/ml) into the right SN at a flow rate of 0.2 μl/min. Animals were PFA-perfused 2 wk after virus injection. 40-μm thick serial coronal sections from the SN were collected on a cryostat (Leica) and processed for immunohistochemistry.

### MPTP injections and stereology in mice

MPTP injections [20] and quantification of dopaminergic neurodegeneration [47] was performed as previously described. Counting of TH- and Nissl-positive neurons in the substantia nigra was performed blind to avoid biases. For statistical analyses, a two-way ANOVA followed by a Newman–Keuls post hoc test was performed (R software packages, version 2.8.0, R Development Core Team 2008, Vienna, Austria).

### Immunohistochemistry of mouse brain sections

Mice were transcardially perfused with 50 ml in a phosphate buffer followed by 100 ml 4% PFA in PB at a flow rate of 15 ml/min. Brains were removed, postfixed overnight in the same solution, then cryoprotected by immersion in 30% sucrose for 36 h. Free-floating 40 μm serial coronal sections from throughout the SN were collected with a Cryostat (Leica Microsystems, Germany). Brain slices were placed in a phosphate-buffered saline solution (1x PBS, 0.1% sodium azide) and stored at 4°C before use.

For the detection of proteins after infection, free-floating sections were washed three times in PBS, blocked in PBS containing 4.5% bovine saline albumin (BSA) for 1 h, then incubated for 48 h at 4°C in PBS containing 3% BSA, 0.2% Triton X-100, and one of the following antibodies of interest. Sections were rinsed three times in PBS and incubated with Alexa Fluor 488-labeled antimouse or rabbit IgG or Alexa Fluor 555-labeled antimouse or rabbit IgG (Invitrogen) at room temperature for 1 h. The sections were rinsed in PBS and the nuclei were counterstained with DAPI (dilution 1:10,000, Wako) for 3 min. The sections were mounted in Mowiol.

Series of sections were immunostained for aSyn to localize the injection site in aSyn-injected mice with anti–N-terminal aSyn antibody (Mouse, BD Transduction Laboratories, 610787). Anti-TH antibody (Rabbit, Millipore, ab152) was used to quantify the loss of dopaminergic (DA) neurons in the injected mice.

### Quantification of neurodegeneration

The total number of DA neurons was determined by counting the number of TH-immunoreactive cells by stereology in every sixth (rat) or fourth (mouse) brain section in the region of the SNpc (6–7 sections throughout the SN). Ventral tegmental TH-positive cells were discarded. StereoInvestigator software (MicroBrightField, Bioscience) was used to count cells in an unbiased manner. The estimation of the total number of TH-positive neurons per SNpc was achieved using the optical fractionator method [48]. In rat brain samples, survival of DA neurons was determined at 1, 2, and 3 wk after vector administration. In mouse brain samples, survival was analyzed 2 wk after vector administration.

## Supporting information

S1 Fig Variation of aSyn acetylation levels with ageing. Brain protein extracts of WT and T2.KO young (2 month) and old (8 month) mice were probed for acetyl-lysine (red) and aSyn (green) (n = 4 per group). The ratio of acetyl-lysine/aSyn ratio is presented. **p < 0.01, **** p < 0.0001, ordinary one-way ANOVA followed by Tukey’s multiple comparisons test. Data in S1 Data.xls.
(TIF)

S2 Fig aSyn KxK motifs are less susceptible to chemical acetylation in vitro. Peptide mass spectrometry analysis of chemically acetylated recombinant aSyn, showing the number of acetylation occurrences. Each green bar represents a detected peptide, and a red dash indicates an acetylation.
(TIF)

S3 Fig Acetylation-resistant aSyn mutant maintains the typical NMR signal and membrane-binding ability. **(A)** Superposition of 2D ^1^H-^15^N HSQC NMR spectra of recombinant ^15^N-labelled aSyn WT (black), K6+10R (red). **(D)** Residue-specific changes in ^1^H-^15^N HSQC signal intensities of aSyn WT (black) and aSyn K6+10R (red) upon addition of SUVs formed by POPC:POPA (1:1 molar ratio). The aSyn-to-lipid molar ratio was 1:100.
(TIF)

S4 Fig Autophagy is impaired upon T2.KD in the cell model of aSyn aggregation. Protein extracts from Scr or T2.KD cells co-transfected with SynT and Synphilin-1 were probed for P62 and β-Actin. Normalized levels of P62 are presented (n = 3). ** p < 0.01, unpaired t-test with equal SD. Data in S1 Data.xls.
(TIF)

S5 Fig aSyn constructs, viral vector details, and experimental paradigm. **(A)** aSyn double mutants mimicking the acetylated (KQ) or the acetylation-resistant (KR) variants of aSyn on K6 and K10. **(B)** Recombinant adeno-associated viral vectors (AAV) serotype 6 expressing green fluorescent protein (GFP), human wild type (WT), KQ, KR variants of aSyn under the control of human synapsin 1 promoter were produced and purified according to standard protocols. **(C)** Young adult female Wistar rats were stereotaxically injected on the right hemisphere (brain coordinates: AP:- 4.7; ML: -2.2; DV: -7.7 mm relative to Bregma to target the SN) with vectors encoding for GFP or aSyn variants. Abbreviations: AP, anterior-posterior; ML, medio-lateral; DV, dentro-ventral.
(TIF)

S6 Fig Acetylation-resistant mutant of aSyn is toxic. Lactate dehydrogenase levels (LDH) were measured in the supernatants of primary cultures infected with AVV6 encoding for WT, KQ or KR aSyn, at different time points after transduction. The KR mutant is toxic 3 days after infection, and the toxicity is then indistinguishable at later time points. Data in all panels are average ± SD, * p < 0.05, two-way ANOVA with Bonferroni correction was used for statistical calculations (n = 4). Data in S1 Data.xls.
(TIF)

S7 Fig Expression of WT aSyn in the SN is toxic over time. **(A)** Brain sections immunostained for TH (red panels) and aSyn (Syn-1) (green panels) 1, 2 and 3 weeks after injection with vectors encoding for EGFP or WT aSyn. Scale bar for isolated channels 200 μm and for merged channels 100 μm. **(B)** Stereological counting of the number of TH-positive neurons in the SN. The EGFP-injected SN of the different groups of animals was used as a control. Statistical comparisons were performed using a one-way ANOVA with Bonferroni multiple comparisons test (*p <0.01, GFP as control; n = 5 animals per condition; six to seven sections from a 1 in 6 series were analysed per brain). Data in S1 Data.xls.
(TIF)

S8 Fig Aggregation pattern of aSyn in the rat substantia nigra. Brain sections immunostained for aggregated-aSyn (red) and GFP or pS129 aSyn (green) from representative animals 3 weeks after injection with AAV6 vectors encoding for GFP and WT, KR or KQ aSyn. **(A)** 5G4 and GFP (GFP group) or pS129 (aSyn groups) merged signal with DAPI is presented. Scale bar 500 μm. **(B) **Higher magnification of the previous groups. Scale bar for isolated channels 50 μm and for merged channels 25 μm.
(TIF)

S1 Table aSyn acetylation in mouse brain, identified by peptide mass fingerprint (PMF) (trypsin digestion). Theoretical peptide mass (Da); error (ppm); Start-end identified peptides; peptide sequences; putative acetylation residues. Oxid (M), N-terminal acetylation and acetylation (K) as variable modifications.
(DOCX)

S1 Data Raw data. Results from Figs 1F, 2B, 2F, 3A, 3B, 4A, 4B, 4C, 5C, 6B, 7B, 7E, 7F, S1, S4, S6 and S7B.
(XLSX)

## Abbreviations

AAV: adeno-associated virus
ALP: autophagy lysosome pathway
aSyn: α-synuclein
CHX: cycloheximide
DIV3: day in vitro 3
EGFP: enhanced GFP
GFP: green fluorescent protein
HEK: human embryonic kidney cells
ICC: immunocytochemistry
IP: immunoprecipitated
LDH: lactate dehydrogenase
MAP2: microtubule-associated protein 2
MS: mass spectrometry
MW: molecular weight
NMR: nuclear magnetic resonance
PD: Parkinson disease
Scr: scramble shRNA
shRNA: short hairpin RNA
SIRT: sirtuin
SN: substantia nigra
SUV: small unilamellar vesicle
TH: tyrosine hydroxylase
ThT: thioflavin T
T2.KD: SIRT2 knockdown
T2.KO: SIRT2 knockout
WT: wild type
