# Correction: The mechanism of sirtuin 2-mediated exacerbation of alpha-synuclein toxicity in models of Parkinson disease

**DOI:** 10.1371/journal.pbio.1002601

**Published:** 2017-04-05

**Authors:** Rita Machado de Oliveira, Hugo Vicente Miranda, Laetitia Francelle, Raquel Pinho, Éva M. Szegö, Renato Martinho, Francesca Munari, Diana F. Lázaro, Sébastien Moniot, Patrícia Guerreiro, Luis Fonseca-Ornelas, Zrinka Marijanovic, Pedro Antas, Ellen Gerhardt, Francisco Javier Enguita, Bruno Fauvet, Deborah Penque, Teresa Faria Pais, Qiang Tong, Stefan Becker, Sebastian Kügler, Hilal Ahmed Lashuel, Clemens Steegborn, Markus Zweckstetter, Tiago Fleming Outeiro

The eleventh author’s name is incorrect. The correct name is: Luis Fonseca-Ornelas.
