# Supplementary figures and images for: The mechanism of sirtuin 2–mediated exacerbation of alpha-synuclein toxicity in models of Parkinson disease
Source: PLoS Biol. 2017 Mar 3;15(3):e2000374. doi: 10.1371/journal.pbio.2000374 (PMC5336201; doi:10.1371/journal.pbio.2000374)

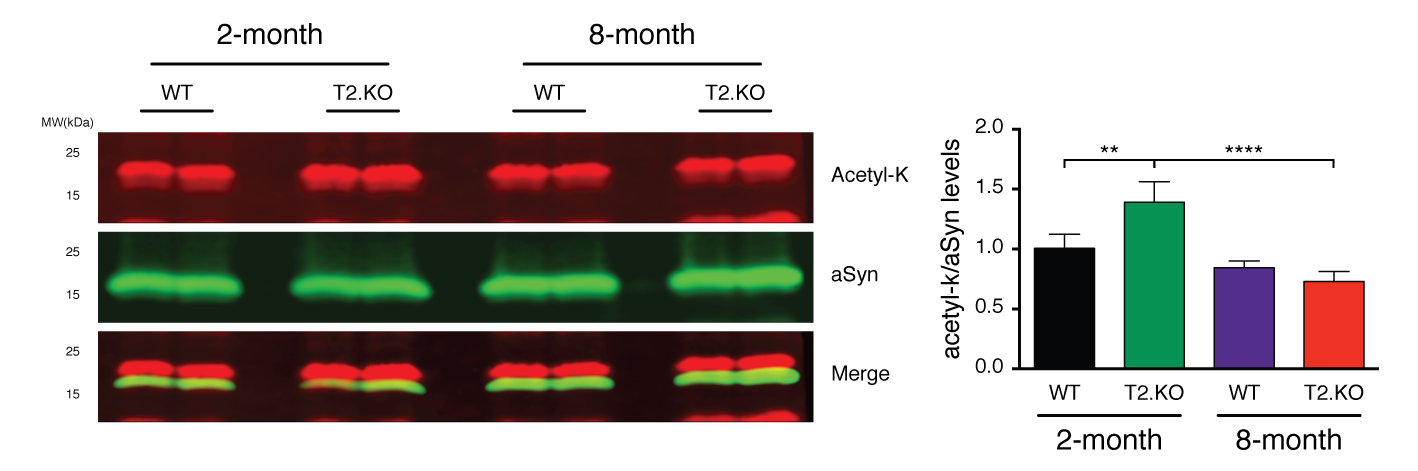

Supplement: S1 Fig — Brain protein extracts of WT and T2.KO young (2 month) and old (8 month) mice were probed for acetyl-lysine (red) and aSyn (green) (n = 4 per group). The ratio of acetyl-lysine/aSyn ratio is presented. **p < 0.01, **** p < 0.0001, ordinary one-way ANOVA followed by Tukey’s multiple comparisons test. Data in S1 Data.xls. (TIF) [file pbio.2000374.s001.tif]

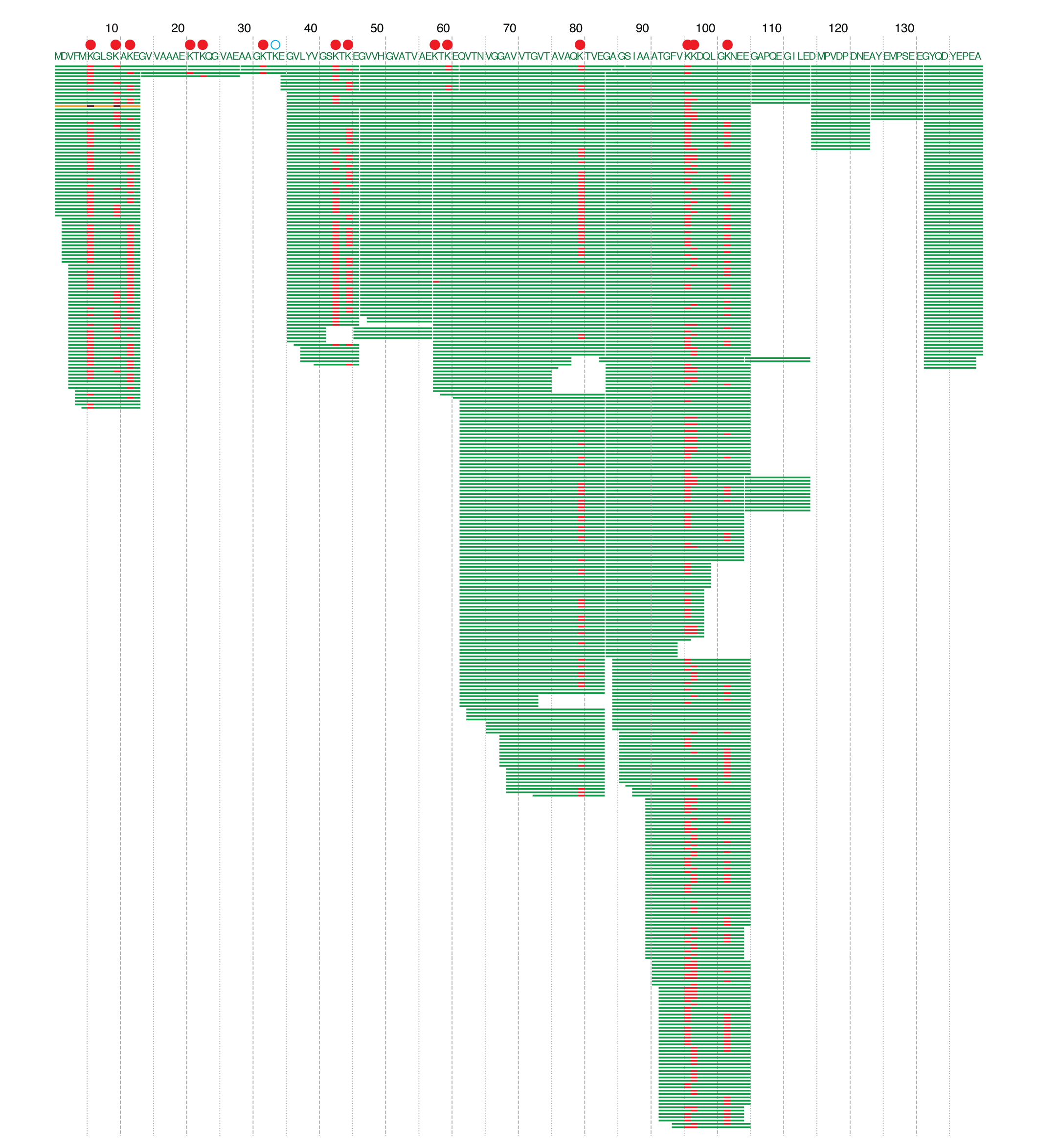

Supplement: S2 Fig — Peptide mass spectrometry analysis of chemically acetylated recombinant aSyn, showing the number of acetylation occurrences. Each green bar represents a detected peptide, and a red dash indicates an acetylation. (TIF) [file pbio.2000374.s002.tif]

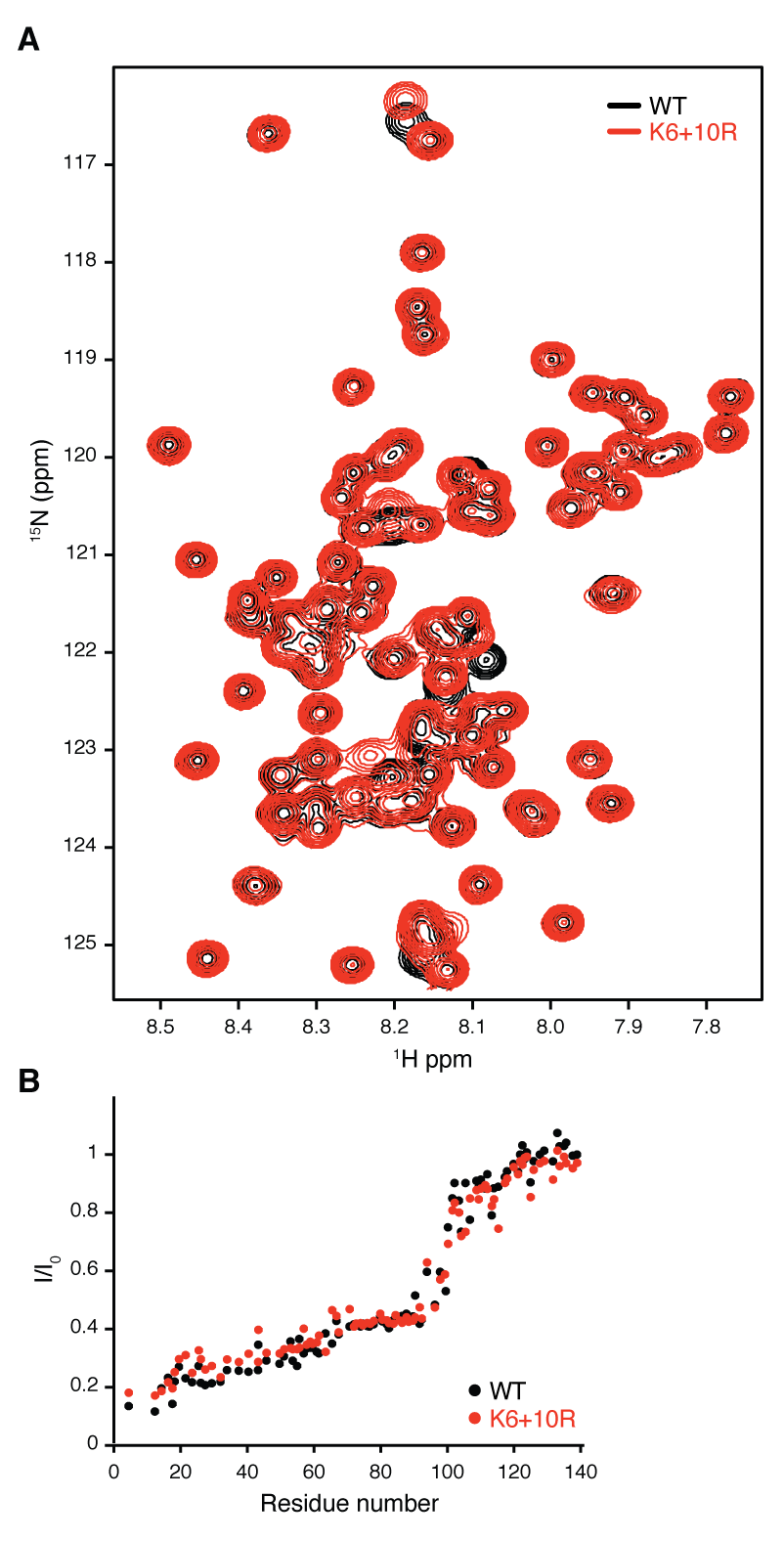

Supplement: S3 Fig — (A) Superposition of 2D 1H-15N HSQC NMR spectra of recombinant 15N-labelled aSyn WT (black), K6+10R (red). (D) Residue-specific changes in 1H-15N HSQC signal intensities of aSyn WT (black) and aSyn K6+10R (red) upon addition of SUVs formed by POPC:POPA (1:1 molar ratio). The aSyn-to-lipid molar ratio was 1:100. (TIF) [file pbio.2000374.s003.tif]

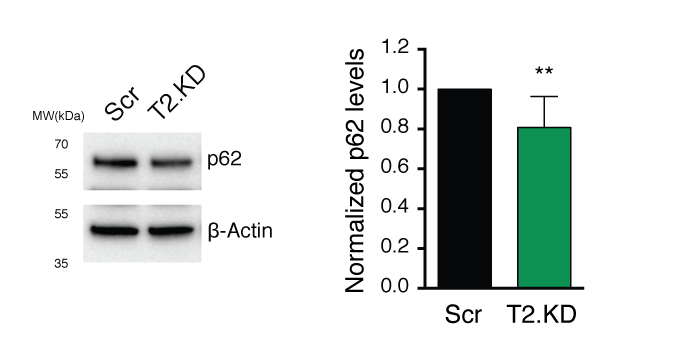

Supplement: S4 Fig — Protein extracts from Scr or T2.KD cells co-transfected with SynT and Synphilin-1 were probed for P62 and β-Actin. Normalized levels of P62 are presented (n = 3). ** p < 0.01, unpaired t-test with equal SD. Data in S1 Data.xls. (TIF) [file pbio.2000374.s004.tif]

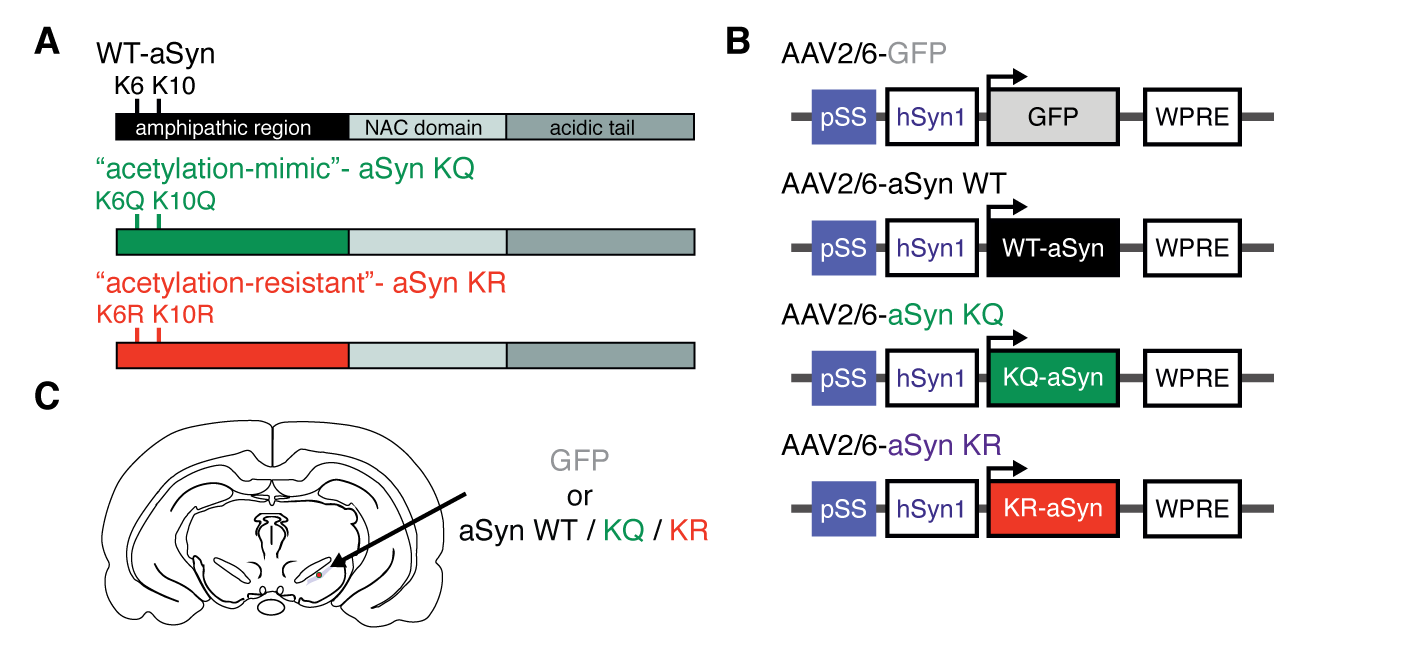

Supplement: S5 Fig — (A) aSyn double mutants mimicking the acetylated (KQ) or the acetylation-resistant (KR) variants of aSyn on K6 and K10. (B) Recombinant adeno-associated viral vectors (AAV) serotype 6 expressing green fluorescent protein (GFP), human wild type (WT), KQ, KR variants of aSyn under the control of human synapsin 1 promoter were produced and purified according to standard protocols. (C) Young adult female Wistar rats were stereotaxically injected on the right hemisphere (brain coordinates: AP:- 4.7; ML: -2.2; DV: -7.7 mm relative to Bregma to target the SN) with vectors encoding for GFP or aSyn variants. Abbreviations: AP, anterior-posterior; ML, medio-lateral; DV, dentro-ventral. (TIF) [file pbio.2000374.s005.tif]

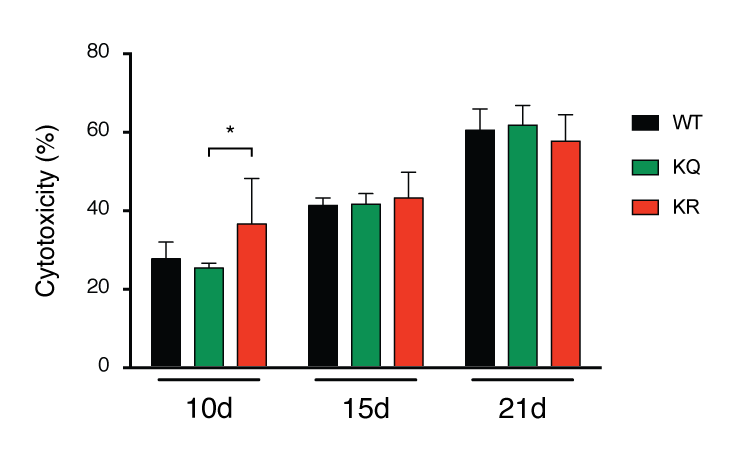

Supplement: S6 Fig — Lactate dehydrogenase levels (LDH) were measured in the supernatants of primary cultures infected with AVV6 encoding for WT, KQ or KR aSyn, at different time points after transduction. The KR mutant is toxic 3 days after infection, and the toxicity is then indistinguishable at later time points. Data in all panels are average ± SD, * p < 0.05, two-way ANOVA with Bonferroni correction was used for statistical calculations (n = 4). Data in S1 Data.xls. (TIF) [file pbio.2000374.s006.tif]

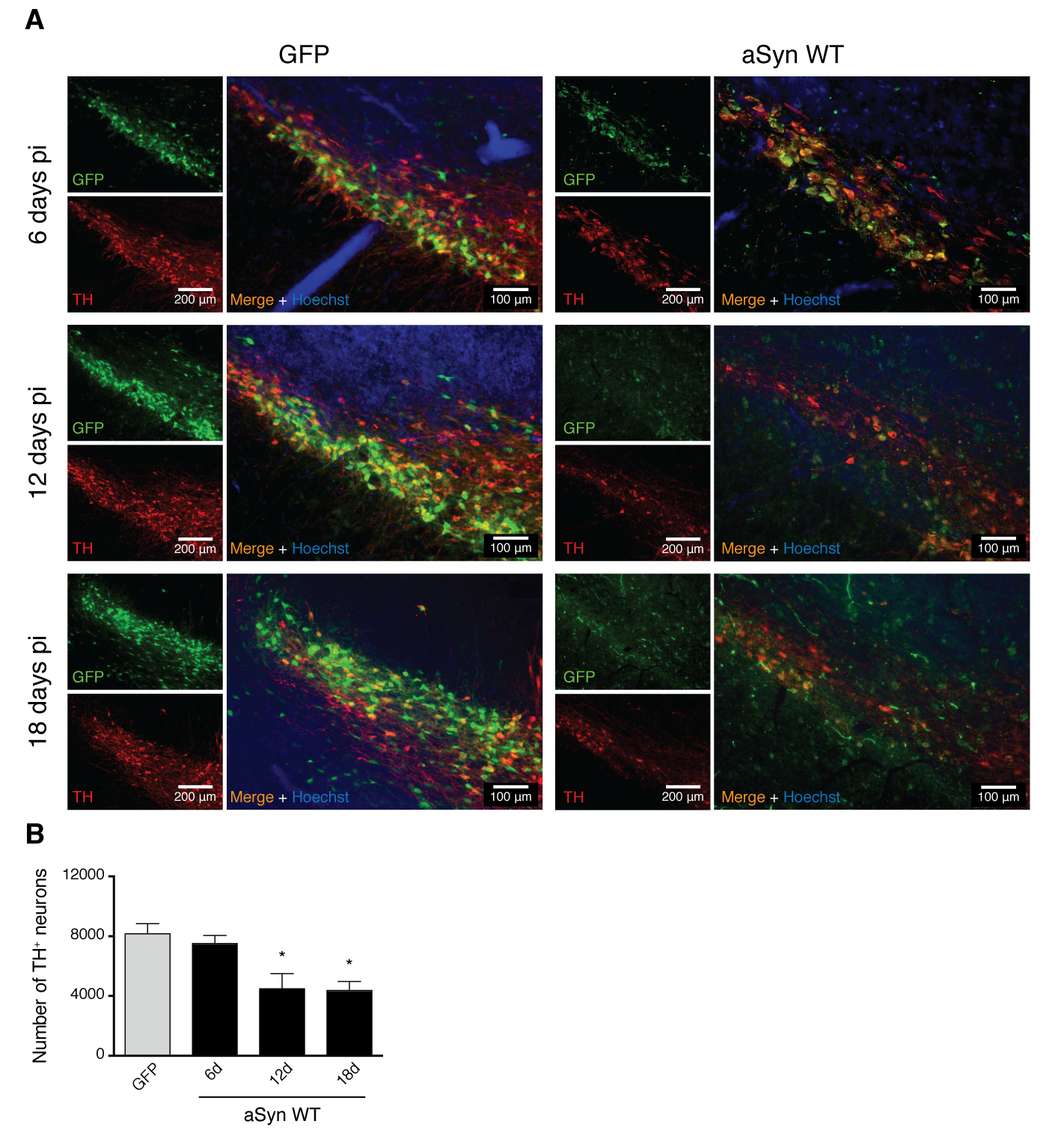

Supplement: S7 Fig — (A) Brain sections immunostained for TH (red panels) and aSyn (Syn-1) (green panels) 1, 2 and 3 weeks after injection with vectors encoding for EGFP or WT aSyn. Scale bar for isolated channels 200 μm and for merged channels 100 μm. (B) Stereological counting of the number of TH-positive neurons in the SN. The EGFP-injected SN of the different groups of animals was used as a control. Statistical comparisons were performed using a one-way ANOVA with Bonferroni multiple comparisons test (*p <0.01, GFP as control; n = 5 animals per condition; six to seven sections from a 1 in 6 series were analysed per brain). Data in S1 Data.xls. (TIF) [file pbio.2000374.s007.tif]

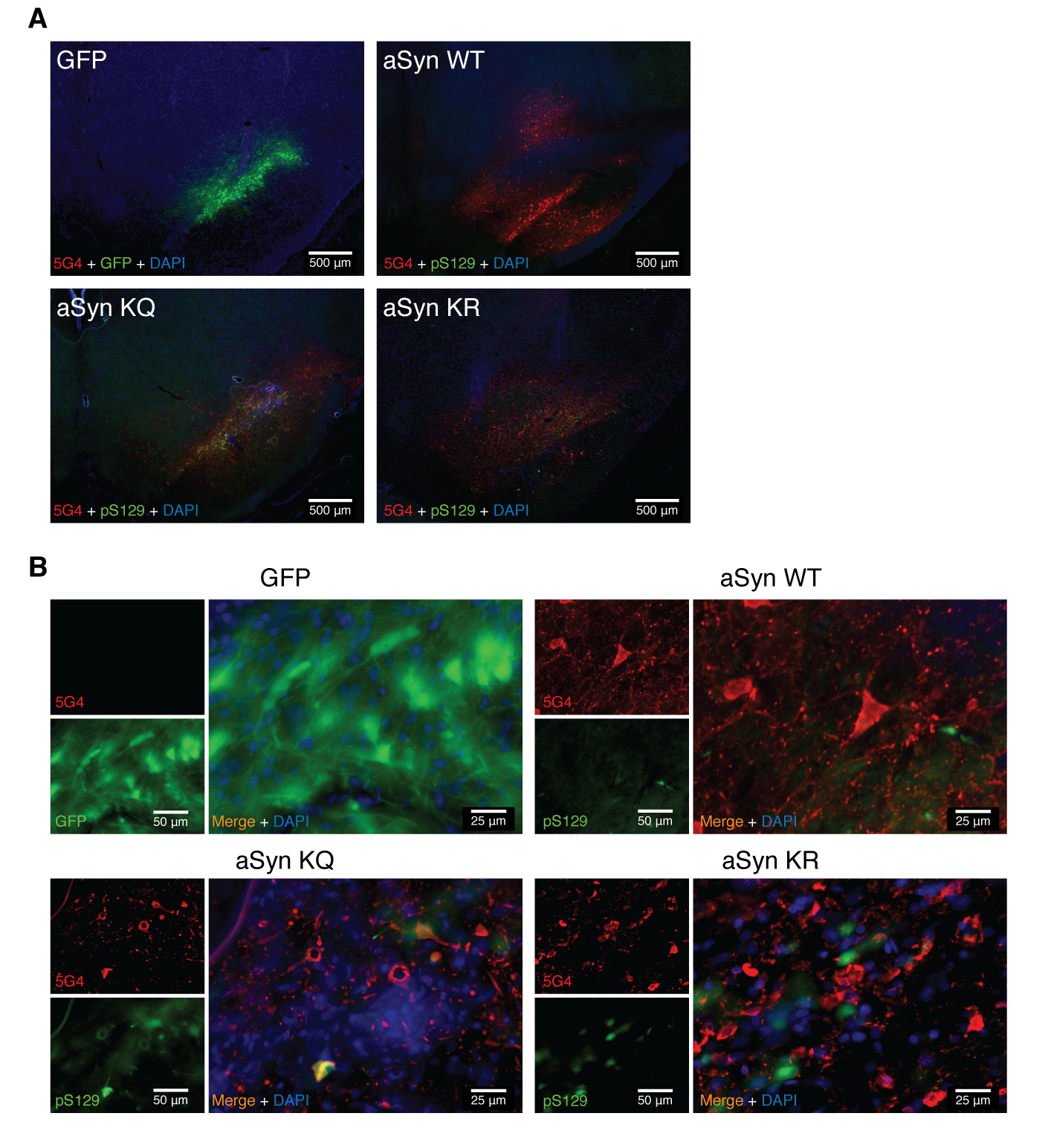

Supplement: S8 Fig — Brain sections immunostained for aggregated-aSyn (red) and GFP or pS129 aSyn (green) from representative animals 3 weeks after injection with AAV6 vectors encoding for GFP and WT, KR or KQ aSyn. (A) 5G4 and GFP (GFP group) or pS129 (aSyn groups) merged signal with DAPI is presented. Scale bar 500 μm. (B) Higher magnification of the previous groups. Scale bar for isolated channels 50 μm and for merged channels 25 μm. (TIF) [file pbio.2000374.s008.tif]
