# Supplementary material for: The mechanism of sirtuin 2–mediated exacerbation of alpha-synuclein toxicity in models of Parkinson disease
Source: PLoS Biol. 2017 Mar 3;15(3):e2000374. doi: 10.1371/journal.pbio.2000374 (PMC5336201; doi:10.1371/journal.pbio.2000374)
Supplement: S1 Table — Theoretical peptide mass (Da); error (ppm); Start-end identified peptides; peptide sequences; putative acetylation residues. Oxid (M), N-terminal acetylation and acetylation (K) as variable modifications. (DOCX) [file pbio.2000374.s009.docx]

| **Theoretical peptide mass (Da)** | **Deviation (ppm)** | **Peptide Sequence** | **Peptide sequence and modifications** | **Putative Acetylated Residues** |
| --- | --- | --- | --- | --- |
| 812.3681 | -3.6 | [1-6] | MDVFMK [1xAcetyl] | N-term |
| 828.363 | -2.3 | [1-6] | MDVFMK [1xAcetyl; 1xOxidation] | N-term |
| 1229.5905 | 7.2 | [1-10] | MDVFMKGLSK [1xAcetyl; 2xOxidation] | K6 |
| 1239.6112 | 82.1 | [1-10] | MDVFMKGLSK [2xAcetyl] | N-term + K6 |
| 1438.7433 | -22 | [1-12] | MDVFMKGLSKAK [2xAcetyl] | K6 + K10 |
| 2225.1668 | -1.5 | [1-21] | MDVFMKGLSKAKEGVVAAAEK [1xOxidation] |  |
| 645.393 | 0.7 | [7-12] | GLSKAK [1xAcetyl] | K10 |
| 1072.5997 | 1.6 | [11-21] | AKEGVVAAAEK |  |
| 873.4676 | -7 | [13-21] | EGVVAAAEK |  |
| 1059.5793 | 1.6 | [22-32] | TKQGVAEAAGK |  |
| 1059.5793 | 1.6 | [24-34] | QGVAEAAGKTK |  |
| 1180.6572 | 6.5 | [33-43] | TKEGVLYVGSK |  |
| 951.5146 | -4.9 | [35-43] | EGVLYVGSK |  |
| 1180.6572 | 6.5 | [35-45] | EGVLYVGSKTK |  |
| 1554.8486 | 8.1 | [44-58] | TKEGVVHGVTTVAEK |  |
| 3693.0181 | 0 | [44-80] | TKEGVVHGVTTVAEKTKEQVTNVGGAVVTGVTAVAQK |  |
| 1325.706 | 7.3 | [46-58] | EGVVHGVTTVAEK |  |
| 1554.8486 | 8.1 | [46-60] | EGVVHGVTTVAEKTK |  |
| 2157.1874 | 6.2 | [59-80] | TKEQVTNVGGAVVTGVTAVAQK |  |
| 1928.0447 | 8.1 | [61-80] | EQVTNVGGAVVTGVTAVAQK |  |
| 1505.7958 | 8.7 | [81-96] | TVEGAGNIAAATGFVK |  |
| 1633.8908 | 10.7 | [81-97] | TVEGAGNIAAATGFVKK |  |
| 722.3502 | -32.9 | [97-102] | KDQMGK [1xOxidation] |  |
| 578.2603 | 24.1 | [98-102] | DQMGK |  |
| 594.2552 | 0.2 | [98-102] | DQMGK [1xOxidation] |  |
| 4827.9543 | 51.8 | [98-140] | DQMGKGEEGYPQEGILEDMPVDPGSEAYEMPSEEGYQDYEPEA [2xOxidation] |  |
